# Breaking Kasha's rule for photoswitchable reactive oxygen species generation in carbonylated carbon nitride

**DOI:** 10.1093/nsr/nwaf062

**Published:** 2025-02-21

**Authors:** Jun Zhao, Hui Li, Zhihao Li, Shenlong Jiang, Manqin Guan, Peng Zhang, Shu Shang, Zhi Zhao, Hui Wang, Qun Zhang, Xiaodong Zhang, Yi Xie

**Affiliations:** Hefei National Research Center for Physical Sciences at the Microscale, University of Science and Technology of China, Hefei 230026, China; Department of Chemical Physics, University of Science and Technology of China, Hefei 230026, China; Hefei National Research Center for Physical Sciences at the Microscale, University of Science and Technology of China, Hefei 230026, China; Hefei National Laboratory, University of Science and Technology of China, Hefei 230088, China; Hefei National Research Center for Physical Sciences at the Microscale, University of Science and Technology of China, Hefei 230026, China; Hefei National Research Center for Physical Sciences at the Microscale, University of Science and Technology of China, Hefei 230026, China; Hefei National Research Center for Physical Sciences at the Microscale, University of Science and Technology of China, Hefei 230026, China; Hefei National Research Center for Physical Sciences at the Microscale, University of Science and Technology of China, Hefei 230026, China; Hefei National Research Center for Physical Sciences at the Microscale, University of Science and Technology of China, Hefei 230026, China; Hefei National Research Center for Physical Sciences at the Microscale, University of Science and Technology of China, Hefei 230026, China; Department of Chemical Physics, University of Science and Technology of China, Hefei 230026, China; Hefei National Laboratory, University of Science and Technology of China, Hefei 230088, China; Hefei National Research Center for Physical Sciences at the Microscale, University of Science and Technology of China, Hefei 230026, China; Hefei National Research Center for Physical Sciences at the Microscale, University of Science and Technology of China, Hefei 230026, China

**Keywords:** photoswitchable, anti-Kasha, molecular-oxygen activation, singlet oxygen

## Abstract

Photoswitchable catalysis enables the non-invasive regulation of light-to-chemical energy conversion, in which catalysts with wavelength-dependent photoexcitation behaviors are typically required. However, for semiconductors, the ultrafast hot-excited-species cooling, depicted as Kasha's rule, tends to induce the accumulation of photoinduced species on the lowest excited states, which brings about a detrimental effect on achieving photoswitchable catalysis. Herein, by taking polymeric carbon nitride as an example, we demonstrate photoswitchable molecular-oxygen activation that is achieved by incorporating carbonyl groups into the matrix: in the range of 300–400 nm, hydroxyl radical and singlet oxygen are identified as the dominant reactive oxygen species under high- and low-energy excitations, respectively. Photoluminescence and ultrafast transient absorption measurements confirm the breakdown of Kasha's rule in carbonylated carbon nitride in which the obstructed relaxation of hot excited species leads to nontrivial wavelength-dependent photophysical properties. This anti-Kasha behavior is also attributed to carbonyl-induced spin–orbit coupling in which the mixing of singlet and triplet states would lead to robust leaps/relaxations between these states, which in turn affect the cooling process of hot photoinduced species. This work deepens the understanding of modulating hot-excited-species relaxation for gaining versatile semiconductor-based photocatalysis.

## Teaser

Excitation-wavelength-dependent photocatalytic oxygen activation has been successfully achieved in a polymeric carbon nitride system, where the breakdown of Kasha’s rule induced by carbonyl-group incorporation was demonstrated to be responsible.

## INTRODUCTION

Photocatalysis provides an intriguing approach to light-to-chemical energy conversion and holds great potential in fields such as environmental engineering [1,2], biomedicine [3,4] and synthetic chemistry [5,6]. Photocatalytic performance is closely related to various photophysical processes that are involved in catalytic systems [7,8] and the corresponding regulation has always been an important topic in the pursuit of advanced photocatalysis. Among a variety of regulation strategies, the excitation-light control of photophysical processes in photocatalysis has recently drawn much attention [9–11]. Compared with the well-explored structural engineering methods, the excitation-light-controlled strategy enables reversible and non-invasive regulation of the photophysical processes of photocatalytic systems and switchable photocatalytic energy conversion could be easily achieved by manipulating the excitation-light wavelength. Apparently, catalysts with energy-dependent photoexcitation properties are typically required for photoswitchable catalysis.

To date, most research on photoswitchable catalysis has been focused on molecular systems including azobenzene [12,13], divinyl [14] and triene systems [15] in which behaviors such as isomerization and 6π electrocyclization play crucial roles in realizing energy-dependent photoexcitation properties and hence photoswitchable catalysis. In comparison, semiconductor-based systems with notable advantages of recyclability and chemical stability seem to hold quite limited potential and only a few cases have been reported to manifest photoswitchable photocatalytic behaviors [16,17]. On the one hand, the semiconductor-based systems possess much higher structural rigidity than the molecular systems and the resulting excellent photostability leads to the infeasibility of photochemical changes under different illuminations. On the other hand, there is a decisive factor against the photoswitchable catalysis of semiconductor-based systems: in contrast to molecular systems, semiconductors tend to have quasi-continuous electronic band structures in which the ultrafast cooling of hot excited species that is mediated by phonon scatterings, depicted as Kasha's rule, tends to induce the accumulation of photoinduced species on the lowest excited state [18–20]. In this case, disrupting hot-excited-species cooling and breaking Kasha's rule should be considered in pursuing excitation-energy-dependent photophysical processes and hence photoswitchable catalysis in semiconductor-based systems.

**Scheme 1.**
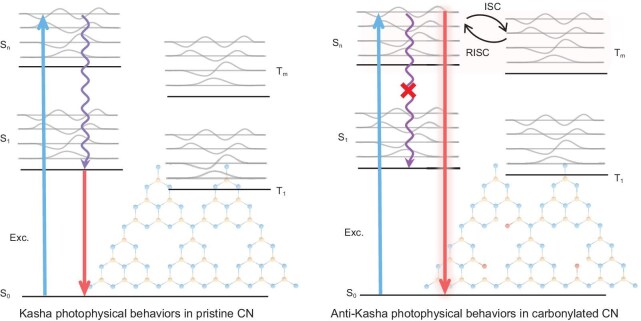
Schematic diagrams of the photophysical processes in pristine and carbonylated CN systems.

Bearing this in mind, we focus our attention on polymeric carbon nitride (CN)—a typical organic semiconductor that has been extensively studied in the field of photocatalysis [21,22] and whose versatile structural and electronic characteristics open the possibility of pursuing photoswitchable catalysis. Owing to its low-dielectric feature, excitons (or bound electron–hole pairs) would be an alternative to free charge carriers dominating the photoexcitation properties of the CN system [23,24], whose kinetics are closely associated with the changes in the spin degree of freedom. That is, as for hot excited species, they would either cool to low-lying excitonic states with the same spin configuration undergoing the internal conversion process or relax to excitonic states with different spin configurations undergoing the intersystem crossing or reverse intersystem crossing processes (Scheme 1). The anti-Kasha behavior is ultimately determined by kinetic factors [25,26], whether it is the modulation of the energy gap [27,28] between the excited states or the spatial distance [29,30], which is about the modulation of the rates of relaxation, internal transitions and radiative processes that are related to photophysical processes. Therefore, as extended semiconductor systems with complex electronic, vibrational and spin–orbit coupling properties, it is possible to modulate the relaxation properties of thermal species by designing characteristic structures to obtain anti-Kasha emission. Given the positive role of carbonyl groups in modifying the electronic, vibrational and spin–orbit couplings between excited states with different spin degrees of freedom [31–36], we implement a carbonylation treatment on pristine CN to modulate the relaxation of hot excited species. Detailed spectroscopic analyses confirm the inhibition of fast hot-excited-species relaxation in carbonylated CN, resulting in anti-Kasha, energy-dependent photoexcitation behaviors. Photocatalytic tests demonstrate photoswitchable molecular-oxygen activation in the system in which hydroxyl radicals and singlet oxygen are the dominant reactive oxygen species under high- and low-energy excitations, respectively.

## RESULTS AND DISCUSSION

**Figure 1.**
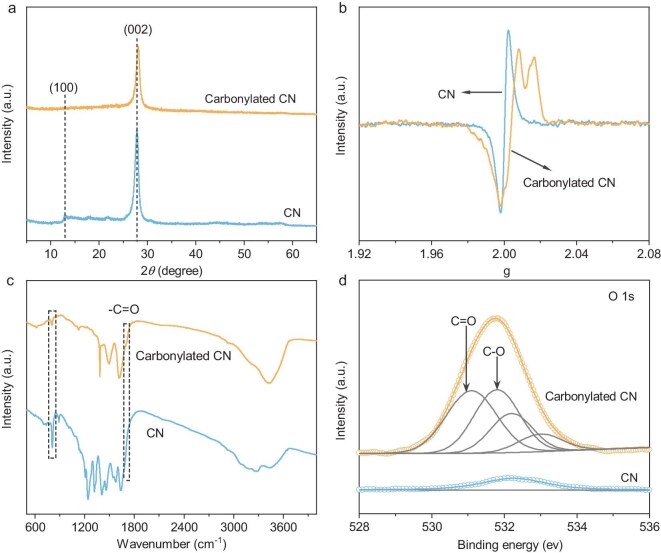
(a) XRD patterns, (b) room-temperature EPR spectra, (c) FT–IR spectra and (d) XPS O 1s spectra of CN and carbonylated CN.

In this study, carbonylated CN was prepared by treating pristine CN with oxidizing acids (see details in Supplementary data). The structure of the samples was first investigated by using X-ray diffraction (XRD) measurements (Fig. 1a). The XRD pattern of CN exhibits two characteristic peaks at 12.9° and 27.8°, corresponding to the (100) and (002) planes, respectively [37,38]. In comparison, the disappearance of the (100) peak and the shift and broadening of the (002) peak for carbonylated CN suggest its weakened periodicity that is induced by the oxidizing-acid treatment. Such a feature agrees well with the results from transmission electron microscopy (Fig. S1) and nitrogen adsorption–desorption isotherms (Fig. S2) in which small broken polymer pieces and a promoted specific surface area were observed after the oxidizing-acid treatment. Figure 1b displays the electron paramagnetic resonance (EPR) spectra of the two samples in which CN exhibits a characteristic EPR signal at ∼2.000 that is assignable to carbon-centered radicals [39,40]. By contrast, carbonylated CN exhibits a superimposed EPR signal, which could be associated with the co-existence of carbon- and oxygen-centered radicals. Besides, compared with that of CN, the carbon-centered radical EPR line of carbonylated CN exhibits obvious broadening, which would be related to the weakened periodicity of the polymer chains. Moreover, the increased oxygen content in carbonylated CN was also confirmed by using element distribution mapping that was acquired through electron probe microanalysis (EPMA) (Fig. S3). Fourier transform infrared (FT–IR) spectroscopy also showed the presence of oxygen-containing groups in carbonylated CN (Fig. 1c). As for the CN sample, characteristic absorption bands at ∼810, 1200–1600 and 3000–3700 cm^−1^ were observed, presenting the breathing mode of triazine units, the stretching vibration of a heterocyclic ring and N–H stretching, respectively [39,41]. As for carbonylated CN, a new band at ∼1720 cm^−1^ emerges, which can be ascribed to the stretching vibration of carbonyl groups [7,42]. Besides, the band for the breathing mode of tri-s-triazine units exhibits an obvious red shift in carbonylated CN, pointing to the introduction of carbonyl groups (Fig. S4). The information of functional groups was further interrogated by using X-ray photoelectron spectroscopy (XPS) (Fig. 1d and Fig. S5). As shown in Fig. 1d, the intensity of the O 1s spectrum for carbonylated CN is much higher than that for CN, suggesting the promoted oxygen-containing species in the former. Detailed peak analyses suggest that four peaks centered at 531.1, 531.8, 532.3 and 533.0 eV emerge in carbonylated CN, corresponding to C=O, C–O, adsorbed water and –NO_2_, respectively [7,43]. In comparison, only a signal related to adsorbed water could be observed in the O 1s spectrum for CN. All the above analyses verified the incorporation of the carbonyl group into the polymeric CN matrix.

**Figure 2.**
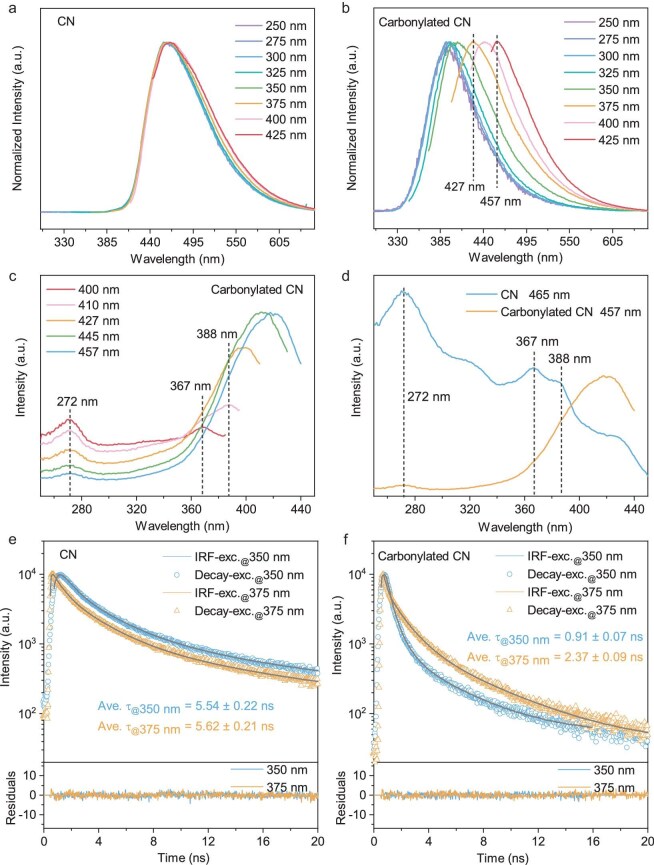
Normalized PL emission spectra of (a) CN and (b) carbonylated CN under different excitation wavelengths. PL excitation spectra were monitored at the corresponding emission peaks of (c) carbonylated CN and (d) CN. Time-resolved PL spectra under 350- and 375-nm excitation (monitored at corresponding emission peaks) of (e) CN and (f) carbonylated CN.

The abundant carbonyl groups induced by the oxidizing-acid treatment are anticipated to impact the cooling of hot photoinduced species. Here, excitation-energy-dependent photoluminescence (PL) measurements were carried out to investigate the involved excited-state properties. According to Ultraviolet-visible (UV-vis) spectra (Fig. S6), a series of excitation wavelengths were selected from 250 to 425 nm with a 25-nm interval. As seen from Fig. 2a, the PL of CN shows a typical Kasha behavior in which the emissions under different excitation wavelengths exhibit quite similar PL spectral lineshapes. Such an excitation-energy-independent feature corresponds to the dominant emissions from the lowest excited state that arose as a result of the rapid cooling of the photoinduced species. Note that the incomplete coincidence of spectral lines at the red end might be related to the slightly different contributions from triplet-exciton emissions. By contrast, obvious excitation-energy-dependent emissions were observed for the carbonylated CN system (Fig. 2b). In detail, excitation wavelengths that were set from 300 to 425 nm (with a 25-nm interval) gave rise to gradually red-shifted emissions that were centered at 394, 398, 405, 427, 442 and 457 nm, respectively. Excitation wavelengths of <300 nm do not lead to variations in the emission spectral lineshape. The spectral-lineshape similarity is primarily attributed to two key factors: first, photoinduced species accommodated at higher-lying excited states tend to possess higher kinetic energies and the resulting more robust electron–electron or/and electron–phonon scatterings would give rise to faster cooling processes toward certain excited states; second, for the region of high-energy excited states, dense superposition of energy levels leads to a reduced energy gap between adjacent excited states, which would also facilitate the cooling of the accommodated hot photoinduced species to certain excited states. The above two factors dominate the cooling of higher-energy excitations, manifesting as similar PL emission spectral lineshapes under 250- to 300-nm excitations. In fact, even in molecular anti-Kasha systems, only finite emission peaks (typically, two or three peaks) [25] could be observed and higher-energy excitations would not give higher-energy emissions. Given the quite complicated structural feature of the CN matrix, it is rather difficult to distinguish the exact originations of these emissions. However, according to the symmetry of emission spectral lineshapes, several characteristic emission bands could be observed. For instance, emission spectra under 375-, 400- and 425-nm excitations exhibit obvious bands at ∼457 nm, whereas emission spectra under 350- and 375-nm excitations exhibit obvious bands at ∼427 nm. Such features imply that the emissions under different excitation wavelengths might consist of several certain bands with different proportions. The deduction could be also evidenced by the excitation-energy-dependent PL spectra of the sample with a higher carbonylation degree (see details in Note S1 and Fig. S7). In this case, the red shift of the emission spectra with the decrease in the excitation energy (from 300 to 425 nm) confirms the suppression of hot-exciton cooling toward the lowest excited states in carbonylated CN.

To identify the origins of anti-Kasha behaviors in carbonylated CN, PL excitation spectra were further recorded. As displayed in Fig. 2c, for carbonylated CN, the excitation spectra of 400-, 410-, 427-, 445- and 457-nm emissions exhibit similar spectral profiles and the dominant peaks that are responsible for these emissions emerge at the same locations, such as ∼272, 367 and 388 nm. These phenomena confirm that all the observed emissions originate from a series of the same excited states. Besides, although exhibiting distinct intensity differences, all these locations in the excitation spectra of carbonylated CN are addressable in that of pristine CN (Fig. 2d). This consistency in the positions of the excitation spectral peaks of CN and carbonylated CN is more pronounced under low-temperature conditions (Fig. S8). The similarities in their excitation spectra verify that carbonylation treatment does not change the original excited-state energy distributions that are involved in the CN matrix. Moreover, the temperature-dependent PL tests could also provide further evidence. Owing to the suppressed electronic–vibrational couplings under low temperatures [44,45], some fine structures could be observed in the PL spectra of carbonylated CN. As shown in Fig. S9, the PL spectra acquired at <30 K exhibit a series of sharp peaks or broad swellings that are located at the same wavelengths of ∼400, 412, 428, 440, 456, 468 and 485 nm. These features could be interpreted as meaning that all of the emissions consist of a series of emissions from specific excited states and excitation energy mainly impacts the proportions of these emissions. The above results provide conclusive evidence that the anti-Kasha characteristics of carbonylated CN are not associated with impurity- or inhomogeneity-related states.

Meanwhile, the fine structures in the emission spectra of carbonylated CN under low temperatures inspire us to interrogate the impacts of electron–phonon couplings on the relevant anti-Kasha characteristics. Regarding this issue, we also measured the temperature-dependent PL emission spectra of CN in which no fine PL structures could be observed for the CN sample under low-temperature conditions (Fig. S10). The different temperature-dependent emission features suggest different vibrational properties between the CN and carbonylated CN samples. To go further, Raman measurements under different additional illuminations were conducted. Note that resonance Raman spectroscopy would be more beneficial for gleaning electron–phonon coupling information, whereas the disturbance from strong PL backgrounds of CN-based systems sets limitations on the relevant applications. Here, a 1064-nm laser was selected as the Raman excitation source and Raman spectra under dark and 320-/380-nm illumination conditions were collected. As shown in Fig. S11, the Raman spectra of CN exhibit several distinct bands within the range of 700–1630 cm^−1^, which are characteristic of graphitic CN [46]. Furthermore, the specific vibrations observed at 753, 977, 1120, 1156, 1236 and 1314 cm^−1^ are indicative of the stretching vibration within the aromatic C–N heterocycles—a feature that is typical of melem [47]. The peaks that fall within the range of 700–1000 cm^−1^ are associated with various ring-breathing modes of s-triazine. Carbonylated CN possesses much weaker Raman spectral intensity than CN in the absence of any additional illumination, which would be associated with the broken structures that are induced during oxidation treatments. Notable new, similar Raman peaks emerge at ∼386, 886, 1241 and 1486 cm^−1^ for both CN and carbonylated CN under additional illuminations. Besides, the intensities of these peaks are distinct under 320- and 380-nm additional illuminations, suggesting the different participations of the corresponding phonons in the two cases. Detailed fitting results confirm the presence of subtle differences between these peaks of CN and carbonylated CN in which peak-value shifts and full width at half maximum (FWHM)-value distinctions could be observed. According to the above Raman results, it could be concluded that carbonylated CN possesses similar electron–phonon coupling to the CN sample and those subtle changes (including peak-value shifts and FWHM differences) do not seem to be responsible for the anti-Kasha characteristics of carbonylated CN.

Particle sizes might influence factors such as surface unsaturated bonds, adsorption properties and packing environments [48], which inevitably execute impacts on photophysical processes. We are inspired to estimate the potential effect of particle size on the relevant PL properties. By using centrifugation under different speeds, we obtained carbonylated CN samples with various particle-size distributions (Fig. S12) and observed their excitation-energy-dependent fluorescence emissions (Fig. S13). Besides, the emission spectral profiles of different samples under the same excitation wavelengths remain unchanged (Fig. S14). This suggests that the anti-Kasha photophysical behavior is not determined by the particle size of carbonylated CN (see details in Note S2). Besides, to pinpoint the principal functional groups behind the excitation-energy-dependence that is observed in carbonylated CN, we conducted targeted treatments to selectively remove specific functional groups. The carbonyl-group-eliminated sample exhibited faint emission variation with excitation energy (see detailed discussions in Note S3 and Figs S15 and S16). Then, *in situ* XPS and EPR measurements under different excitations were carried out to gain insights into the mechanism associated with the anti-Kasha behaviors of carbonylated CN. Figure S17 displays the XPS O 1s spectra that were acquired under dark, 320- and 380-nm illuminations. Compared with dark conditions, 320- and 380-nm illuminations led to notable but different changes in spectral factors including FWHM and peak values, implying that oxygen-containing species are involved in relevant photoexcitation processes. A more detailed fractionation treatment of the peaks shows the different contributions of oxygen-containing functional groups under the corresponding illuminations. Taking the scenario of C=O species as an example, the peak position is almost unchanged under 380-nm excitation but is shifted toward higher binding energy under 320-nm excitation. For C–O, the peak position is also almost unchanged under 380-nm excitation but is shifted toward lower binding energy under 320-nm excitation. These would be associated with the different electronic excited-state configurations that are involved under different excitations. Similar results could be obtained according to the excitation-energy-dependent EPR results. As shown in Fig. S18, illumination could lead to the increase in the EPR signal for both CN and carbonylated CN samples, owing to the trapping of photoinduced electrons at certain sites. For carbonylated CN, the ratio of carbon- to oxygen-centered radical signals exhibits a notable excitation-energy-dependent feature. In detail, the peak shapes of EPR under 380-nm excitation and dark conditions are basically the same, which indicates that the contributions of carbon-centered radicals and oxygen-centered radicals to the photoexcitation process (including charge transfer and/or excitation) of the system are similar. In contrast, under the 320-nm excitation condition, the peak shapes show obvious changes and the proportion of oxygen-centered radicals decreases, which is likely to be related to the electron transfer from oxygen-containing species to carbon-containing species. These EPR features clearly verify the different participation scenarios of oxygen-containing functional groups under different excitations. The above results provide direct evidence of the crucial role of oxygen-containing functional groups in achieving anti-Kasha behaviors in carbonylated CN. Based on the above results, it can be assumed that the positive role of the carbonyl group in promoting spin–orbit coupling [33–36] makes the mixing of singlet and triplet states, and leads to robust leaps/relaxations between these states, thus affecting the cooling process of hot photoinduced species. This is believed to be the main reason for the anti-Kasha property of carbonylated CN.

Furthermore, time-resolved PL measurements were carried out to investigate the radiative decay kinetics under different excitation wavelengths. A tri-exponential function was employed to fit the PL decay kinetics due to the better residuals compared with those of bi-exponential fittings. As shown in Fig. 2e, time-resolved PL spectra under 350- and 375-nm excitations for the CN sample exhibit quite similar decay profiles with a nearly identical average PL lifetime (i.e. ∼5.54 and 5.62 ns); besides, the statistical weights of the decay components under 350- and 375-nm excitations are also quite close (Table S1). The similar decay kinetics correspond to the rapid cooling of hot excitons and the emission from the lowest excited states under different excitation wavelengths. In stark contrast, there are significant variations between the decay kinetics under different excitation wavelengths for carbonylated CN (Fig. 2f) in which faster decay can be detected under the higher excitation energy. The average PL lifetimes are ∼0.91 and 2.37 ns for 350- and 375-nm excitation scenarios, respectively. Note also that the decay-component statistical weights under 350- and 375-nm excitations of carbonylated CN are distinctly different (Table S1). This phenomenon is in accordance with the fact that high-energy excited states tend to possess shorter lifetimes. All the above results provide confirmative evidence of the nontrivial, excitation-energy-dependent photophysical properties in carbonylated CN in which the crucial role of carbonyl groups in modulating the cooling of hot excitons is identified. Moreover, to confirm the generality of carbonylation-induced anti-Kasha behaviors in CN systems, we prepared two other control samples under a different synthesis temperature (denoted CN-1) and with a different precursor (denoted CN-2). The CNs that were synthesized by using with different precursors and different synthesis methods had similarities but different structures and different degrees of polymerization [49], which may affect the PL properties. Carbonylated CN-1 and carbonylated CN-2 were obtained by using the same acid treatment and they also showed excitation-energy-dependent emission properties (see details and discussions in Note S4, Figs S19–S28 and Tables S2 and S3).

**Figure 3.**
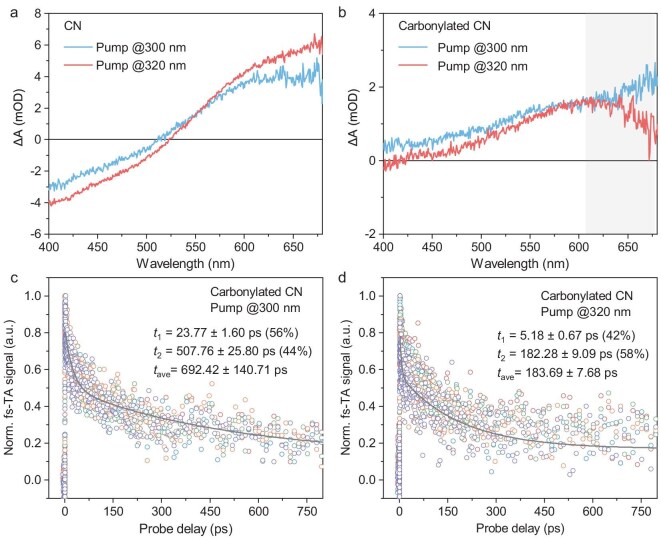
Representative fs-TA spectra taken at a 2-ps probe delay under 300- and 320-nm excitations for (a) CN and (b) carbonylated CN. The corresponding kinetic traces excited at (c) 300 nm and (d) 320 nm for carbonylated CN.

To further understand the excitation-energy-dependent excited-state behaviors in carbonylated CN, femtosecond time-resolved transient absorption (fs-TA) measurements with a pump–probe configuration were conducted. Here, pump wavelengths were set at 300 or 320 nm due to superior optical absorption and signal-to-noise ratio, and a white-light (400–680 nm) probe was selected to monitor the corresponding fs-TA spectral changes. Figure 3a and b display the representative fs-TA spectra that were taken at the 2-ps probe delay under 300-/320-nm excitation for pristine CN and carbonylated CN, respectively. For pristine CN, both probe-bleach and -absorption signals within the monitoring range could be observed, which are attributed to singlet excitons and free charges, respectively [50,51]. Note that similar spectral lineshapes under the two pump wavelengths can be interpreted as similar excited-state energy profiles that are related to the rapid cooling of hot photoinduced species. In contrast, 300- and 320-nm excitations gave rise to obvious spectral variations in the red end (shadowed window in Fig. 3b) for carbonylated CN, hinting at different cooling scenarios and transient populations of high-energy excited species under the two excitations. Note that the observation of faint probe-bleach signals across the whole monitoring range for carbonylated CN might be associated with the low emission intensities under high-energy excitations (Fig. S29). The different excited-state behaviors between pristine CN and carbonylated CN were also evidenced by the subtle differences in the relaxation kinetics under certain excitations. As shown in Fig. S30, decay traces under 300- and 320-nm excitations for pristine CN exhibit similar tendencies. The similar time constants that were derived from a bi-exponential global fitting (five traces with a 5-nm interval in 620–640 nm) suggest the pump-wavelength-independent excited-state relaxation in CN, which was commensurate with the results of PL measurements. For carbonylated CN (Fig. 3c and d), 320-nm excitation turned out to give a faster decay than 300-nm excitation, which clearly demonstrates the distinctly different relaxation scenarios under the two excitations. By combining the results from PL measurements, it is safe to conclude that the cooling of hot photoinduced species is obstructed in carbonylated CN, which endows the system with nontrivial excitation-energy-dependent excited-state properties.

**Figure 4.**
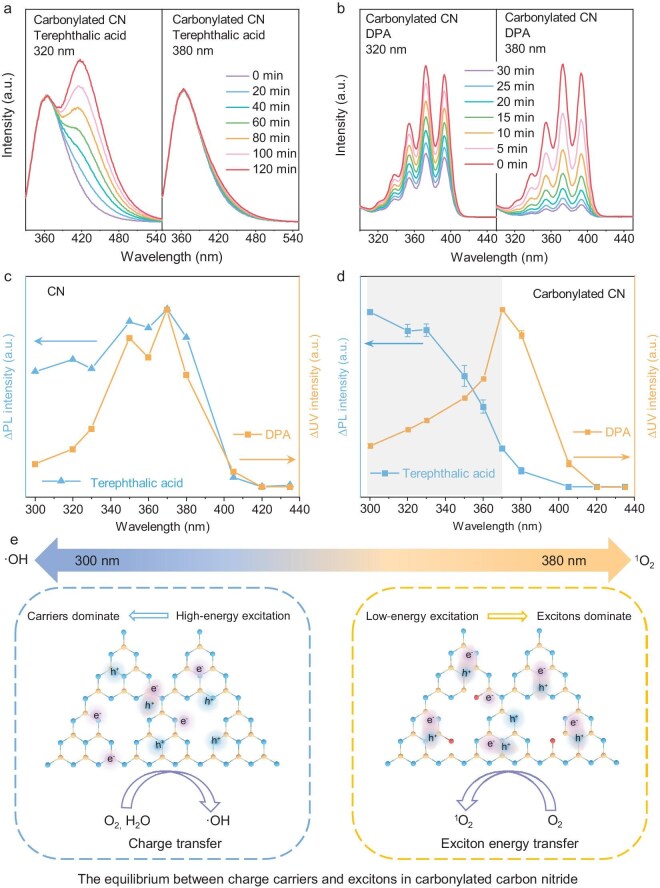
(a) Terephthalic acid measurements for •OH detection and (b) DPA measurements for ^1^O_2_ detection at 320 and 380 nm for carbonylated CN. Wavelength-dependent terephthalic acid and DPA measurements for (c) CN and (d) carbonylated CN by monitoring the PL and UV intensity increments of terephthalic acid (within 60 min) and DPA solutions (within 15 min), respectively. (e) The excitation-energy-dependent photocatalytic behavior of carbonylated CN under high- and low-energy excitations.

By taking advantage of the nontrivial excited-state properties in carbonylated CN, we anticipate modulating the selectivity of photocatalytic reactive oxygen species (ROS) by manipulating the excitation-light wavelength. Here, terephthalic acid and 9,10-diphenyl anthracene (DPA) were employed as molecular indicators to detect photocatalytic •OH and ^1^O_2_ generation. Terephthalic acid and 9,10-diphenylanthracene selectively react with •OH and ^1^O_2_, leading to the formation of 2-hydroxyterephthalic acid and 9,10-diphenylanthracene endoperoxide (Fig. S31), respectively. The corresponding products could be easily monitored by using PL and UV–vis spectroscopies. Figure 4a displays the time evolutions of the PL intensity of terephthalic acid solutions in the presence of carbonylated CN under 320- and 380-nm illuminations. Compared with the faint PL changes for the 380-nm scenario, the remarkable enhancement in PL intensity for the 320-nm scenario clearly verified the suitable •OH generation of carbonylated CN under a high-energy excitation. By contrast, UV–vis absorption evolutions of DPA solutions, as shown in Fig. 4b, revealed that 380-nm illumination is more beneficial to ^1^O_2_ generation for carbonylated CN than 320-nm illumination. Moreover, the reverse •OH and ^1^O_2_ generation under 320- and 380-nm illuminations for carbonylated CN was found to be closely related to carbonyl groups (see details in Note S5 and Fig. S32). For pristine CN, the corresponding measurements (Fig. S33) suggest that 380-nm illumination favors both •OH and ^1^O_2_ generation. To gain a more intuitive understanding of the impact of excitation energy on the relevant photocatalytic ROS generation, wavelength-dependent measurements were further carried out. As displayed in Fig. 4c, the generation of both •OH and ^1^O_2_ for the pristine CN case exhibits similar tendencies across the whole range in which obvious rising edges between 400 and 370 nm can be observed. However, for carbonylated CN, the rising edge of •OH generation exhibits an obvious shift toward the high energy range compared with that of ^1^O_2_ generation (Fig. 4d). In detail, •OH generation increases with the photon energy of the excitation over the full range, whereas ^1^O_2_ generation reaches its maximum at 370 nm and gradually decreases below that wavelength. The opposite tendencies in •OH and ^1^O_2_ generation for carbonylated CN confirm the feasibility of modulating photocatalytic ROS selectivity by controlling the excitation scenario. We also conducted structural characterizations and PL tests on the carbonylated CN samples after photoreactions. The carbonylated CN exhibits significant structural stability during the photoreactions. Notably, the excitation-energy-dependent PL emission features of carbonylated CN are consistently maintained throughout the entire photoreactions (see details in Note S6 and Figs S34–S37).

Considering that various mechanisms are involved for both •OH and ^1^O_2_ generation [52,53], we employed isotope-labeling experiments to identify the scenarios in the carbonylated CN system by using ^18^O-labeled O_2_ and H_2_O as precursors. Detailed results demonstrate that molecular-oxygen- and water-related conversion are responsible for ^1^O_2_ and •OH generation, respectively (see details in Note S7 and Figs S38 and S39). Furthermore, the valence-band maximum of carbonylated CN, as derived from UV absorption spectroscopy and Mott-Schottky curve analysis, is determined to be 2.62 eV (vs. normal hydrogen electrode (NHE)). This value exceeds the redox potential of the H_2_O/•OH (2.38 V, vs. NHE), which allows hole-mediated water oxidation for •OH generation (see details in Note S8 and Fig. S40). We rationalize the different photocatalytic behaviors of pristine CN and carbonylated CN as follows. For pristine CN, the similar tendencies in •OH and ^1^O_2_ generation are in accordance with the above-observed wavelength-independent PL behaviors. Owing to the rapid cooling of hot photoinduced species in CN, the lowest-excited-state excitons and charge carriers dominate the PL and photocatalytic behaviors that occur on the nanosecond (or longer) timescale. The equilibrium between the charge carriers and the excitons described by the Saha–Langmuir equation gives rise to similar tendencies in •OH and ^1^O_2_ generation. Note that the deviation between •OH and ^1^O_2_ generation in the wavelength range below 370 nm might be related to hot-carrier-mediated photocatalysis. For carbonylated CN, the opposite tendencies in •OH and ^1^O_2_ generation could be associated with the obstructed cooling of the hot photoinduced species. That is, under a high-energy excitation, the photoinduced species tend to be blocked in high-energy excited states (as evidenced by the PL and fs-TA measurements). Compared with the scenario in low-energy excited states, the equilibrium between the charge carriers and excitons in high-energy excited states would shift toward the charge-carrier side [54,55], thereby resulting in a promoted charge-carrier concentration and a reduced exciton concentration (as shown in Fig. 4e). This phenomenon could be rationalized as follows [56]: hot excitons that are generated under high-energy excitation would quickly relax their excess energies via exciton–phonon scattering; the exchange of thermal energy between the electronic and phonon subsystems would lead to a promoted effective temperature (in detail, lattice temperature) over the conjugation segments of the polymer chains; the elevated temperature favors the exciton dissociation by boosting the charge-transfer states into free carriers or polarons (see details and discussion in Note S9). The photoelectrochemical measurements also verified this (see details in Note S10 and Fig. S41). In addition, excitation density-dependent ROS generation tests for carbonylated CN samples were further performed (see details in Note S11 and Fig. S42). According to the above discussion, promoted •OH generation and suppressed ^1^O_2_ generation could be expected in the high-energy region.

## CONCLUSIONS

In conclusion, we proposed that photoswitchable catalytic behaviors can be achieved by modulating the hot-photo-induced-species cooling processes in semiconductor-based systems. By taking polymeric CN as an example, we demonstrated that the introduction of carbonyl groups improves spin–orbit coupling in which the mixing of singlet and triplet states leads to robust transition/relaxation between these states, thereby affecting the cooling of hot photoinduced species, which confers the system with nontrivial excitation energy-dependent photocatalytic ROS generation. By combining photoluminescence and ultrafast transient absorption spectroscopies, we demonstrated that, in contrast to pristine CN with fast cooling of hot photoinduced species, carbonylated CN exhibits nontrivial anti-Kasha photophysical processes in which the obstructed cooling of the hot photoinduced species results in excitation-energy-dependent excited-state behaviors in the system. Such a feature enables the selectivity of photocatalytic molecular-oxygen activation to be modulated by controlling the excitation energy. That is, in the wavelength region of 300–400 nm, high- and low-energy excitations favor charge-transfer-mediated •OH generation and energy-transfer-mediated ^1^O_2_ generation, respectively. This work establishes a paradigmatic model for pursuing versatile photocatalytic energy utilization via external optical manipulation.

## Supplementary Material

nwaf062_Supplemental_File
