# Supplementary material for: Breaking Kasha's rule for photoswitchable reactive oxygen species generation in carbonylated carbon nitride
Source: Natl Sci Rev. 2025 Feb 21;12(5):nwaf062. doi: 10.1093/nsr/nwaf062 (PMC11983681; doi:10.1093/nsr/nwaf062)
Supplement: nwaf062_Supplemental_File [file nwaf062_supplemental_file.pdf]

Supplementary data

Breaking Kasha's rule for photoswitchable reactive oxygen species generation in carbonylated carbon nitride

Jun Zhao,[1,‡] Hui Li,[2,‡] Zhihao Li,[1] Shenlong Jiang,[3] Manqin Guan,[1] Peng Zhang,[1] Shu Shang,[1] Zhi Zhao,[1] Hui Wang,*[,1] Qun Zhang,*[,1,2,3] Xiaodong Zhang,*[,1] and Yi Xie*[,1]

[1]*Hefei National Research Center for Physical Sciences at the Microscale, University of Science and Technology of China, Hefei, Anhui 230026, China*

[2]*Department of Chemical Physics, University of Science and Technology of China, Hefei, Anhui 230026, China*

[3]*Hefei National Laboratory, University of Science and Technology of China, Hefei, Anhui 230088, China*

*E-mail: wanghuig@ustc.edu.cn; qunzh@ustc.edu.cn; zhxid@ustc.edu.cn; yxie@ustc.edu.cn

[‡]These authors contributed equally to this work.

Experimental section.

Preparations of polymeric carbon nitride (CN), CN-1, CN-2, carbonylated CN, carbonylated CN-1, carbonylated CN-2 and carbonylated CN-80.

CN samples were prepared by polymerization of melamine molecules under high temperature. In detail, 15 g of melamine was put into a crucible (30 mL) with a cover and heated at 600 ℃ for 10 h in a muffle furnace with a ramp rate of 10 ℃ min^{-1} for both the heating and cooling processes. After being cooled down, the obtained yellow product was collected and fully ground, yielding the powder samples denoted pristine CN.

CN-1 samples were prepared by polymerization of melamine molecules under high temperature. In detail, 15 g of melamine was put into a crucible (30 mL) with a cover and heated at 500 ℃ for 10 h in a muffle furnace with a ramp rate of 10 ℃ min^{-1} for both the heating and cooling processes. After being cooled down, the obtained yellow product was collected and fully ground, yielding the powder samples denoted pristine CN-1.

CN-2 samples were prepared by polymerization of dicyandiamide molecules under high temperature. In detail, 15 g of dicyandiamide was put into a crucible (30 mL) with a cover and heated at 550 ℃ for 3 h in a muffle furnace with a ramp rate of 5 ℃ min^{-1} for both the heating and cooling processes. After being cooled down, the obtained yellow product was collected and fully ground, yielding the powder samples denoted pristine CN-2.

The carbonylated CN samples were prepared by oxidation treatment of pristine CN. In detail, 1 g of pristine CN powder was carefully added into the mixture of concentrated sulfuric acid and nitric acid with a volume ratio of 1:3. After being stirred at 60℃ for 8 h, the mixture was poured into 500 mL distilled water and collected by extraction filtration. The white product was washed with distilled water for several times and dried in air at room temperature, yielding the powder samples denoted carbonylated CN. Carbonylated CN-1 and carbonylated CN-2 are obtained by oxidation treatment of CN-1 and CN-2 respectively, and the treatment method is the same. The

carbonylated CN-80 is a more carbonylated sample and which used as a comparison for the emission spectral lineshape, it was synthesized in the same way as the carbonylated CN, except that the stirring temperature was increased to 80 ℃.

Terephthalic acid measurements. In details, 20 mg of carbon nitride sample was added into 50 mL of NaOH (10 mM) and terephthalic acid (3 mM) solution. The mixture was stirred in the dark for 30 minutes before illumination to ensure adsorption/desorption equilibrium between catalyst and indicator. The supernatant was centrifuged at specific time intervals during the light reaction and analyzed for fluorescence testing. For wavelength-dependent experiments, a xenon lamp with a specific wavelength bandpass filter was used as the light source (15 mW/cm^2).

DPA Measurements. 20 mg of carbonylated CN powder and 50 mL of 9,10-diphenylanthracene (0.09 mM) solution were mixed and stirred for 30 min in the dark to reach the equilibrium of adsorption and desorption. Then the light reaction was performed, and a certain volume of the reaction solution was centrifuged at certain time intervals and tested for UV–vis absorption. Band-pass filters with certain wavelengths were used in wavelength-dependent tests, where the light intensities were kept at 15 mW/cm^2. The bleaching of DPA under different excitations should be deducted in dealing with wavelength-dependent data.

Characterization Methods. The TEM images were collected on H-7650 (Hitachi, Japan) operated at an acceleration voltage of 100 kV. The XPS were recorded on a Thermo ESCALAB 250Xi with Al $K\alpha$ ($\hbar\nu$ = 1486.6 eV) as the excitation source. The binding energies derived from the XPS analysis were corrected against the specimen charging by referencing C 1*s* to 284.8 eV. The ultraviolet–visible (UV–vis) spectra were recorded on a Perkin Elmer Lambda 950 UV–vis–NIR spectrophotometer. The nitrogen absorption and desorption curves were recorded on a Tristar II 3020M. Electron probe microanalyses (EPMA) were carried out on EPMA-8050G (Shimadzu,

Japan). The FT-IR spectra were acquired on a Magna-IR750 FT-IR spectrometer in a KBr pellet, scanning from 4000 to 400 cm^{-1} at room temperature. The XRD characterizations were performed on a Philips X'Pert Pro Super diffractometer with Cu Kα radiation (λ = 1.54178 Å). The steady-state and time-resolved PL spectra were obtained on a FLUOROLOG-3-TAU fluorescence spectrometer equipped with an integrating sphere. The fs-TA measurements were performed on a Helios pump–probe spectrometer (Ultrafast Systems LLC) in combination with an amplified fs laser system (Coherent). The pump pulses (0.14 mJ/cm^2 at the sample ） were delivered by an optical parametric amplifier (TOPAS-800-fs). The instrument response function was determined to be ~100 fs. The samples well dispersed in acetonitrile were contained in a 0.7-mL quartz cuvette.

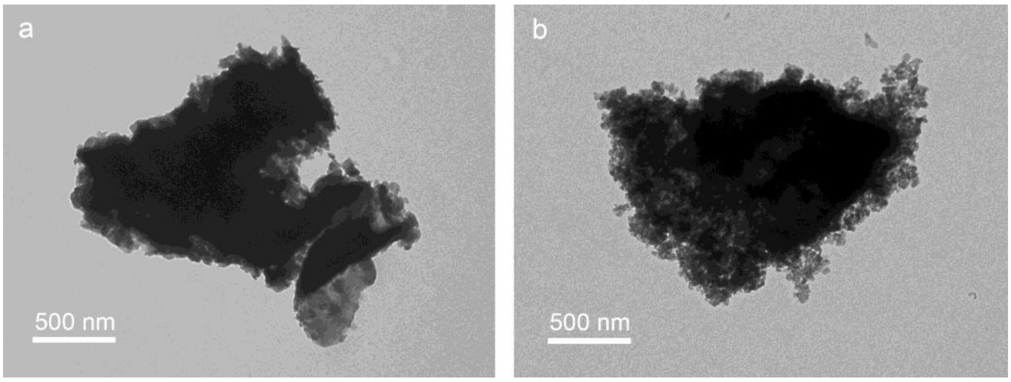

Figure S1. TEM images of (a) CN and carbonylated CN.

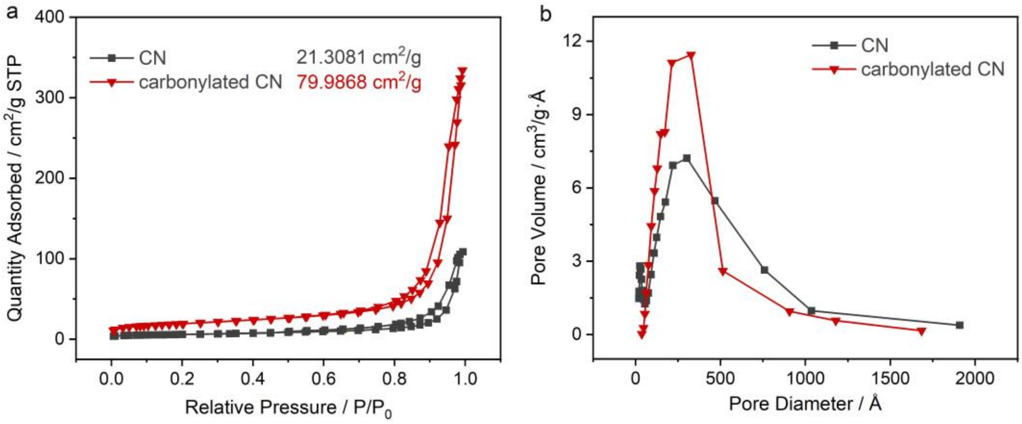

Figure S2. (a) BET and (b) pore-size distribution analyses for CN and carbonylated CN.

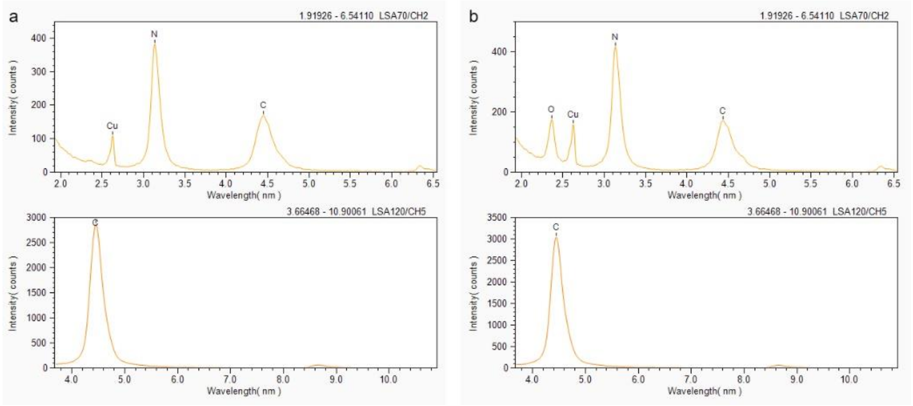

Figure S3. EPMA element analyses of (a) CN and (b) carbonylated CN, from which remarkable promotion in oxygen content can be observed in the carbonylated sample.

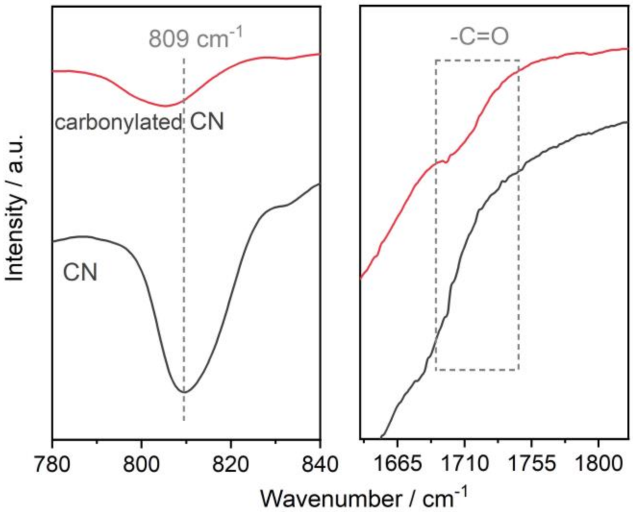

Figure S4. Partial FT-IR spectra of CN and carbonylated CN.

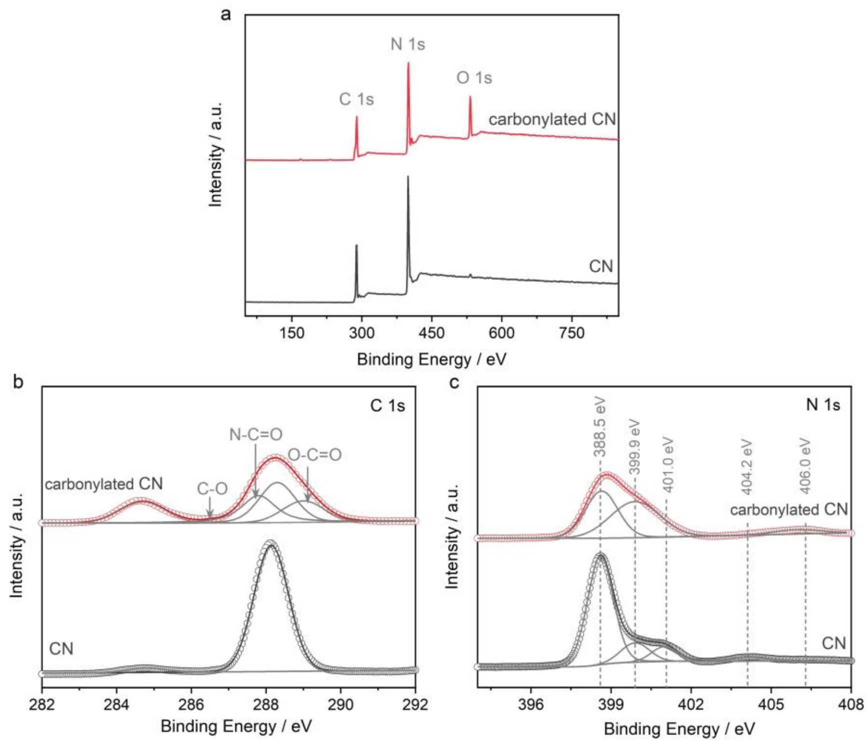

Figure S5. XPS (a) survey spectra, (b) C 1*s* spectra, and (c) N 1*s* spectra of CN and carbonylated CN.

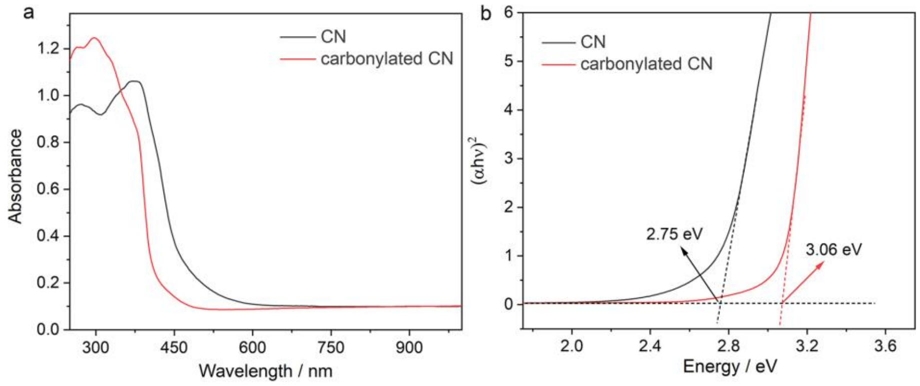

Figure S6. (a) UV–vis spectra and (b) the corresponding Tauc plots of CN and carbonylated CN.

Note S1: Carbonylated CN with higher degree of carbonylation.

Carbonylated CN-80 was synthesized at a higher reaction temperature (80 ℃) and the FT-IR spectra showed a more pronounced peak of C=O stretching vibration (**Figure S7a**), indicating a higher degree of carbonylation. As shown in **Figure S7b**, the photoluminescence of carbonylated CN-80 continues to exhibit excitation-energy-dependent properties. Focus on the lineshapes of the emission spectra, several characteristic emission bands are more evident: the emission spectra at 300-, 325-, 350-, and 375-nm excitations show distinct bands around 404, 427, and 448 nm, further demonstrating that the emission at different excitation wavelengths may consist of several specific bands of different proportions.

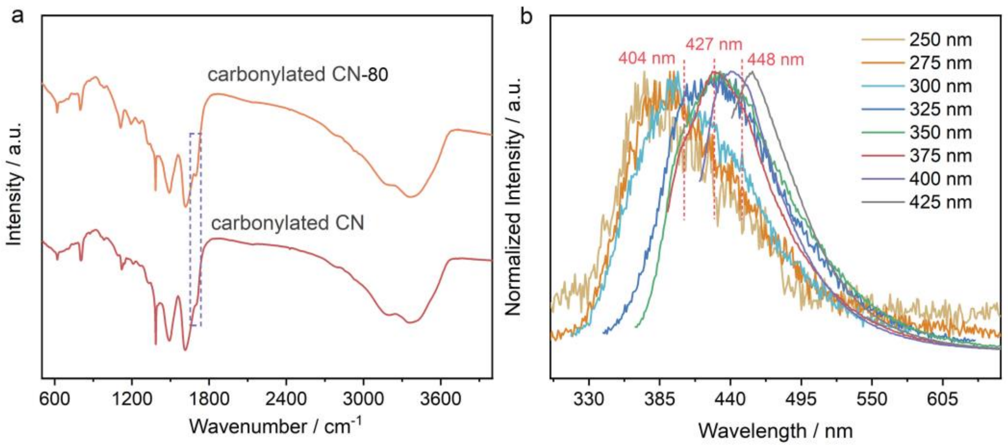

Figure S7. (a) FT-IR spectra of carbonylated CN and carbonylated CN-80 (with more carbonyl groups). (b) Normalized Fluorescence emission spectra of carbonylated CN-80.

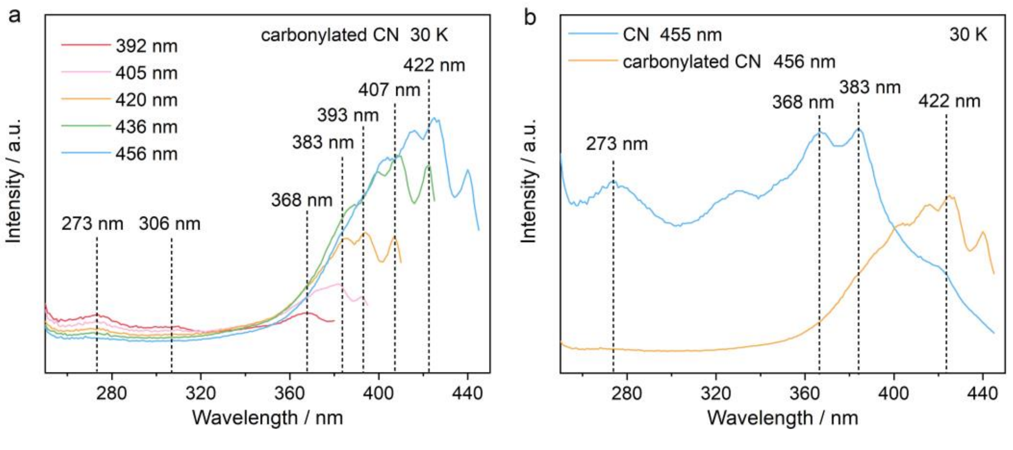

Figure S8. PL excitation spectra of (a) carbonylated CN and (b) CN at 30 K.

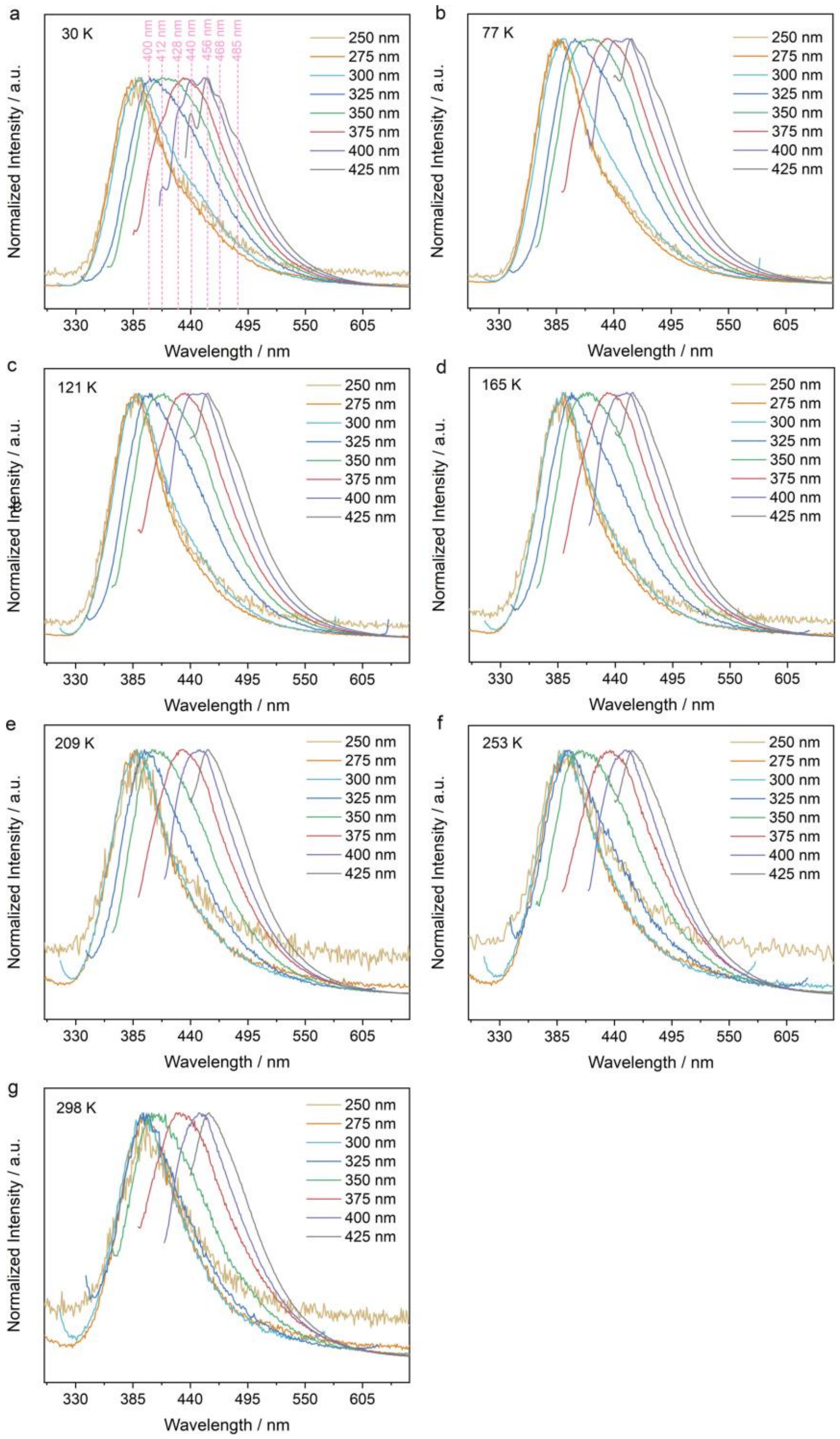

Figure S9. Normalized PL emission spectra of carbonylated CN under different excitation wavelengths at (a) 30 K, (b) 77 K, (c) 121 K, (d) 165 K, (e) 209 K, (f) 253 K, (g) 298 K.

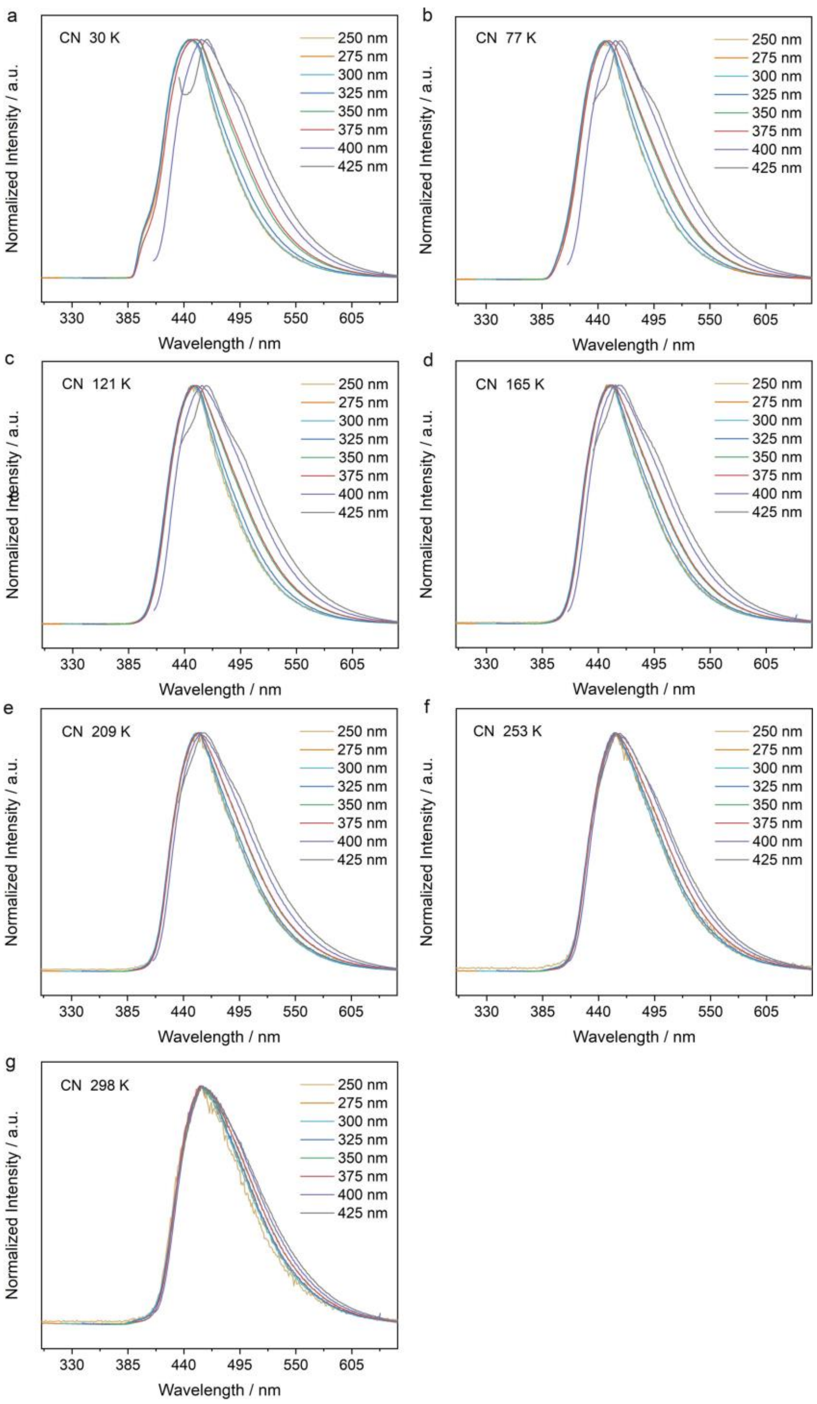

Figure S10. Normalized PL emission spectra of carbonylated CN under different excitation wavelengths at (a) 30 K, (b) 77 K, (c) 121 K, (d) 165 K, (e) 209 K, (f) 253 K, (g) 298 K.

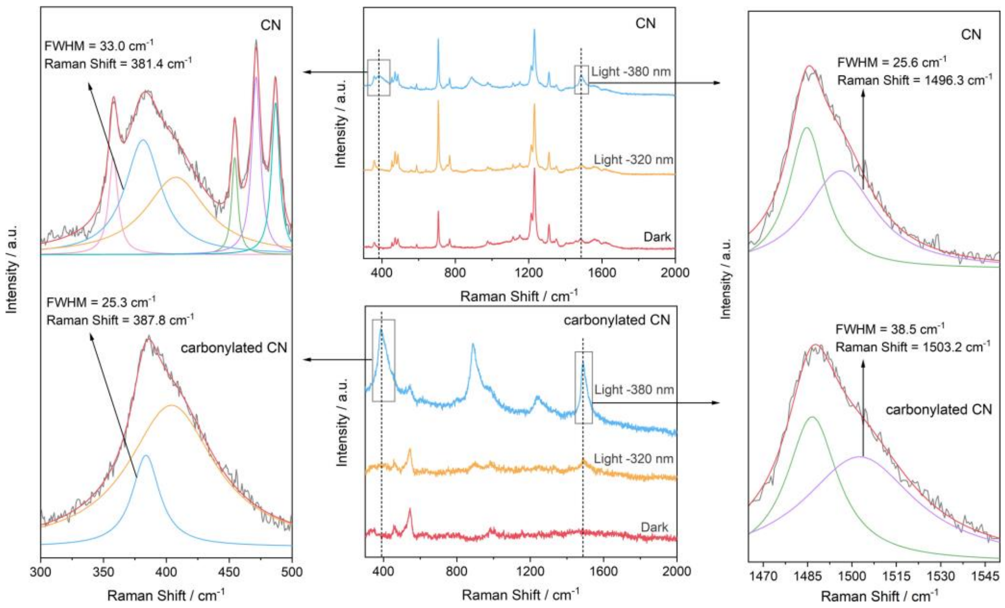

Figure S11. Raman spectra in darkness and light of CN and carbonylated CN.

Note S2: Carbonylated CN with different particle sizes.

Different sizes of material are collected by dispersion of carbonylated CN in water and centrifugation at different speeds. TEM images show significantly different particle size distributions of the samples collected under different centrifugal speeds (**Figure S12**). The fluorescence emission peaks under different excitation light are compared and find to be excitation-energy-dependent for different sizes of carbonylated CN (**Figure S13**), and the positions of the emission peaks are almost constant (**Figure S14**), demonstrating the independence of anti-Kasha photophysical processes on sample size.

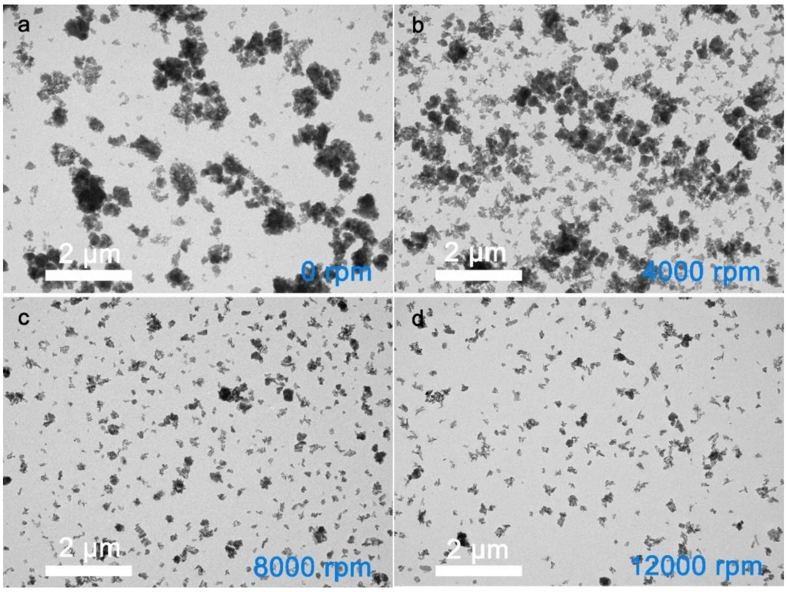

Figure S12. TEM image of carbonylated CN with different size collected at (a) 0 rpm, (b) 4000 rpm, (c) 8000 rpm, (d) 12000 rpm.

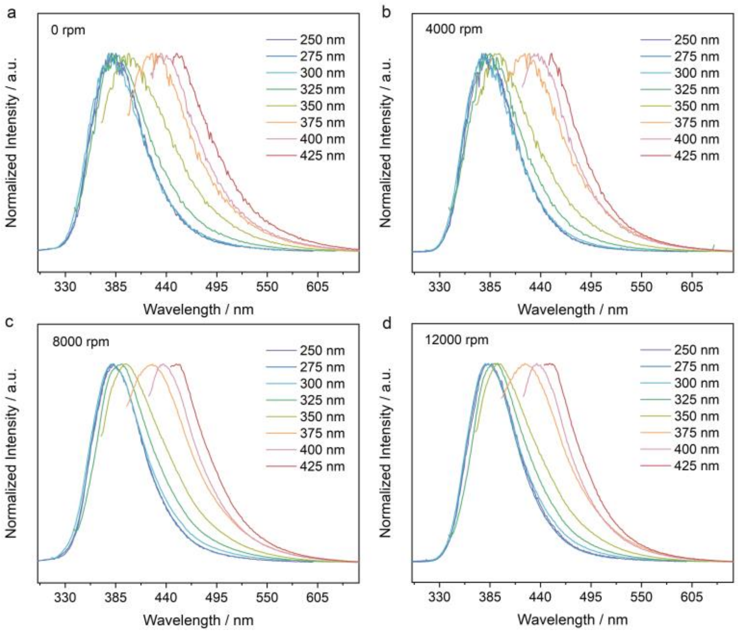

Figure S13. Normalized PL emission spectra of carbonylated CN with different size collected at (a) 0 rpm, (b) 4000 rpm, (c) 8000 rpm, (d) 12000 rpm.

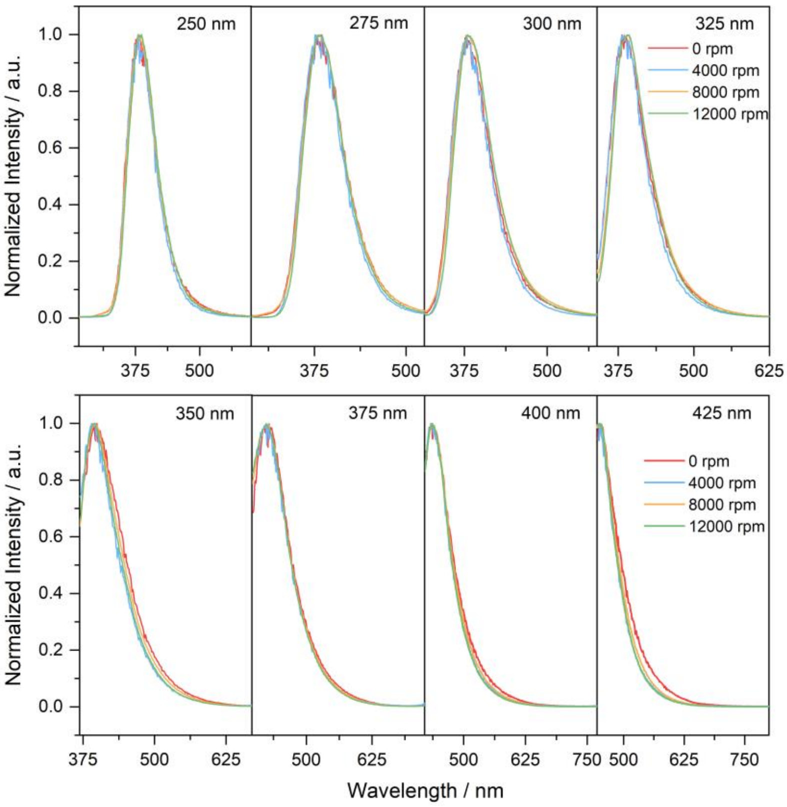

Figure S14. Comparison of normalized PL emission spectra of carbonylated CN with different particle sizes at different excitation wavelengths.

Note S3: Elimination of carbonyl groups and other oxygen-containing functional groups.

During the synthesis of carbonylated CN, the introduction of C=O is accompanied by other oxygen-containing functional groups, including C-O-C, C-O, $-NO_2$, and -OH. To ascertain the primary functional group responsible for the excitation-energy-dependent properties of carbonylated CN, we undertook targeted treatments to remove specific functional groups. Initially, carbonylated CN was annealed under an inert atmosphere to eliminate carbonyl groups. Thermogravimetric analysis shows a significant mass loss of carbonylated CN up to 300 ℃ (**Figure S15**), accounting for the elimination of its carbonyl groups. FT-IR spectroscopy shows the disappearance of the C=O signal after annealing, confirming the successful removal of carbonyl groups (**Figure S16a**). Fluorescence spectroscopy was performed on the reduced-carbonylated CN to monitor the emission behaviors under different excitation wavelengths, and the excitation-energy-dependent emission behaviors nearly disappear embodied in unmovable emission wavelengths of carbonylated CN with increasing excitation wavelengths, expounding this excitation-energy-dependent feature of carbonylated CN is brought by carbonyl groups (**Figure S16b**). Additionally, employing the $HBr/H_2C_2O_4$ treatment[1] to remove epoxy, hydroxyl, and nitro groups from carbonylated CN (**Figure S16c**), did not alter its excitation-energy-dependent traits (**Figure S16d**), indicating that these groups have a negligible effect on the spin-orbit coupling of carbonylated CN.

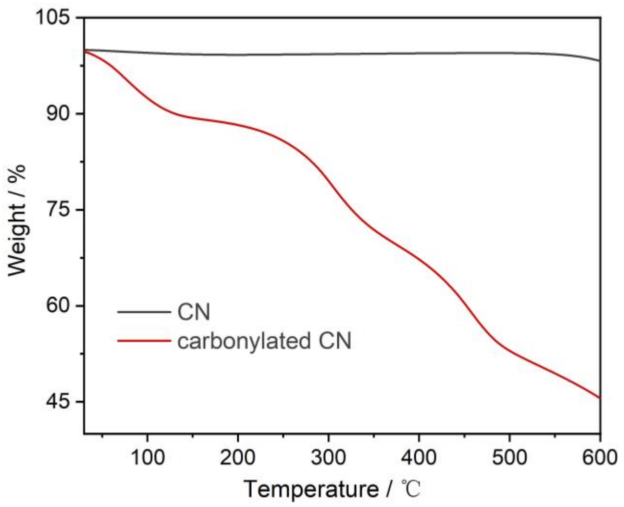

Figure S15. Thermal gravimetry curves of CN and carbonylated CN.

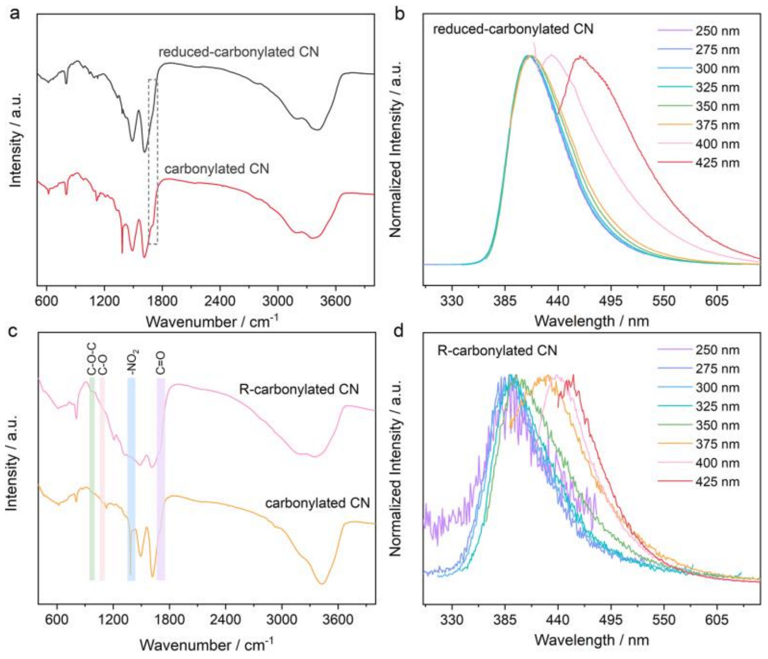

Figure S16. (a) FT-IR spectra of reduced-carbonylated CN and carbonylated CN. (b) Normalized PL emission spectra of reduced-carbonylated CN. Reduced-carbonylated CN is obtained by heating the carbonylated CN to 300 ℃ under an inert atmosphere, which has removed C=O group. (c) FT-IR spectra of R-carbonylated CN and carbonylated CN. (d) Normalized PL emission spectra of R-carbonylated CN. R-carbonylated CN is obtained by employing the $HBr/H_2C_2O_4$ treatment on the carbonylated CN.

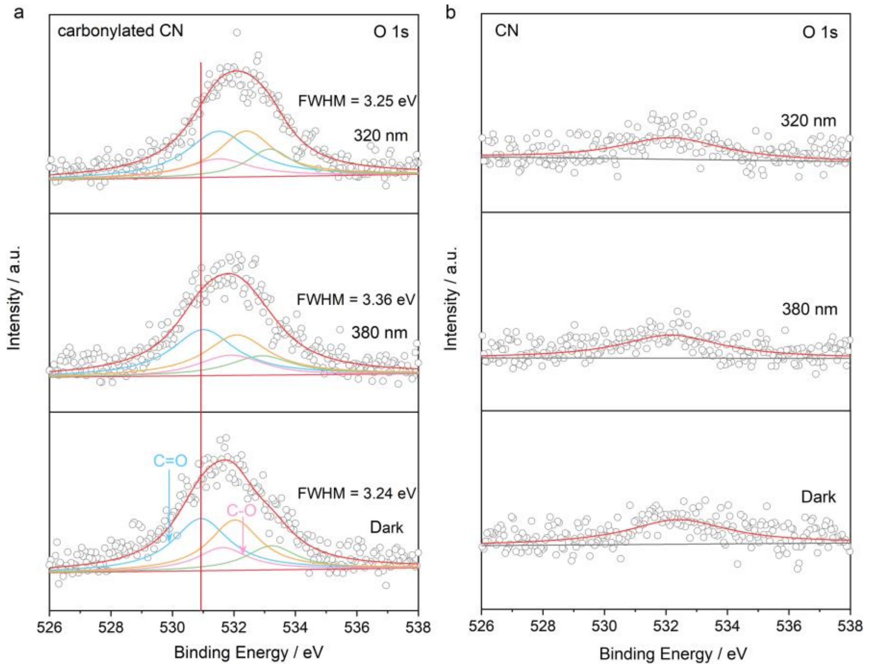

Figure S17. O1s spectra of (a) carbonylated CN and (b) CN under dark, 380nm, 320nm illumination conditions.

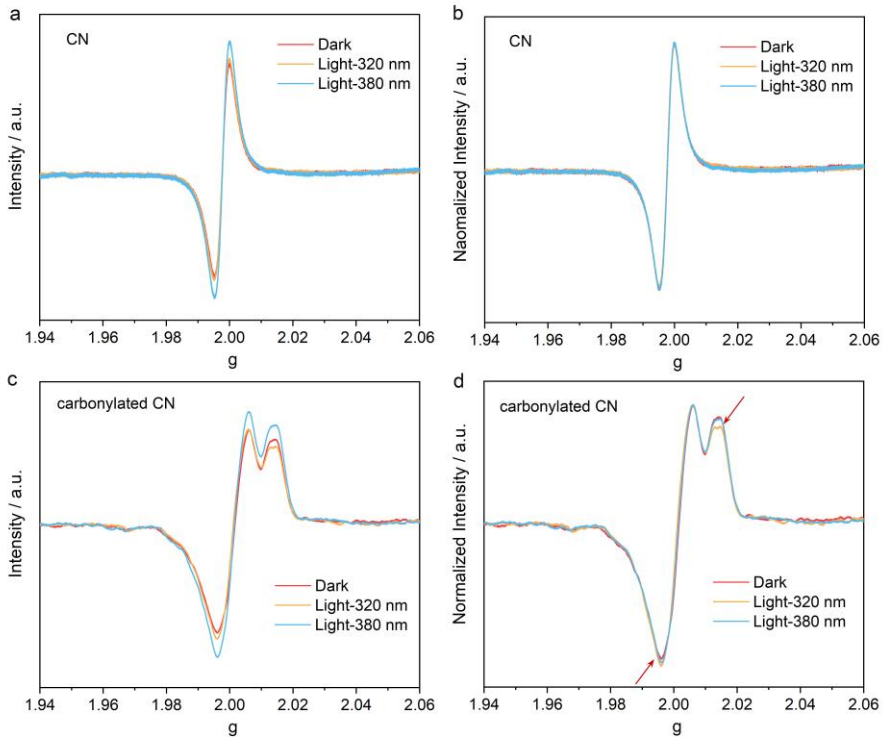

Figure S18. (a) Room temperature EPR spectra and (b) corresponding normalized spectra of CN. (c) Room temperature EPR spectra and (d) corresponding normalized spectra of carbonylated CN.

Table S1. Tri-exponential fitting results of time-resolved PL spectra of CN and carbonylated CN.

Materials	CN		carbonylated CN	
Excitation wavelength	350 nm	375 nm	350 nm	375 nm
T1 / ns	2.59 ±0.18 (44%)	2.50 ±0.15 (44%)	0.78 ±0.10 (16%)	1.21 ±0.09 (45%)
T2 / ns	10.53 ±0.33 (41%)	10.61 ±0.33 (42%)	3.56 ±0.20 (21%)	4.55 ±0.12 (39%)
T3 / ns	0.64 ±0.03 (15%)	0.62 ±0.03 (14%)	0.06 ±0.02 (63%)	0.22 ±0.02 (16%)
Average LifeTime / ns	5.54 ±0.22	5.62 ±0.21	0.91 ±0.07	2.37 ±0.09

Note S4: Discussions related to the control samples CN-1, CN-2, carbonylated CN-1, carbonylated CN-2, in order to confirm the prevalence of carbonylation-induced anti-kasha behavior in carbon nitride systems.

To identify the impact of macroscopic inhomogeneity, we prepared two other control samples under a different synthesis temperature (denoted CN-1) and with a different precursor (denoted CN-2), the experimental details and characterizations are presented as below. It has been widely accepted that carbon nitride samples prepared via different methods possess different inhomogeneities in aspects like degree of polymerization, crystallinity, and surface properties, which would greatly affect their PL properties.[2] In the pore-size distribution plots (**Figure S19a**), the pore volume of CN is much larger than those of CN-1 and CN-2. Besides, the ratios of pore-size distribution are also different, which is related to the differences in their micro-morphological structures. Thermogravimetric analyses (TGA) and differential scanning calorimetry (DSC) studies were utilized to test the thermal stability of CNs samples (**Figure S19b**). The first mass loss from the TGA curves occurring below 200 ℃ is mainly due to volatilization of H_2O or other volatile impurities adsorbed on the sample surface. Further increase of the heating temperature leads to the decomposition of CN, which chemically converts carbon nitride into carbon and nitrogen containing gases. Thermal degradation of CN, CN-1, CN-2 occurs in the temperature ranges of 500–728, 523–738, and 521–728 ℃, respectively. The DSC results are also shown in **Figure S19b** (presented as dash lines). The exothermic peaks of CN, CN-1, and CN-2 were monitored at 717, 722, and 720 °C, respectively, and it was noted that a sharp peak of heat absorption at 729 °C also appeared in the CN-2 sample. Meanwhile, the different line shapes of the TGA and DSC curves also indicate the different decomposition processes of CNs samples, representing their different crystallinities and polymerization degrees. The luminescence properties of the CNs samples are also different: under the excitation of 350 nm, the fluorescence emission peaks of CN, CN-1, and CN-2 are located at 465, 456, and 457 nm, respectively, and the intensities of CN, and CN-1 are significantly larger than that of

CN-2 (**Figure S19c**). In the corresponding excitation spectra, CN showed five major peaks located at 272, 320-, 367, 388, and 427nm, respectively, whereas only the dominant peaks at 274, 366, and 427 nm were observed in CN-1 and CN-2 (**Figure S19d**). The carbonylated CN, CN-1, and CN-2 obtained after oxidation treatment also showed great variabiliteis in pore size distribution (**Figure S19e**) and TGA - DSC test (**Figure S19f**).

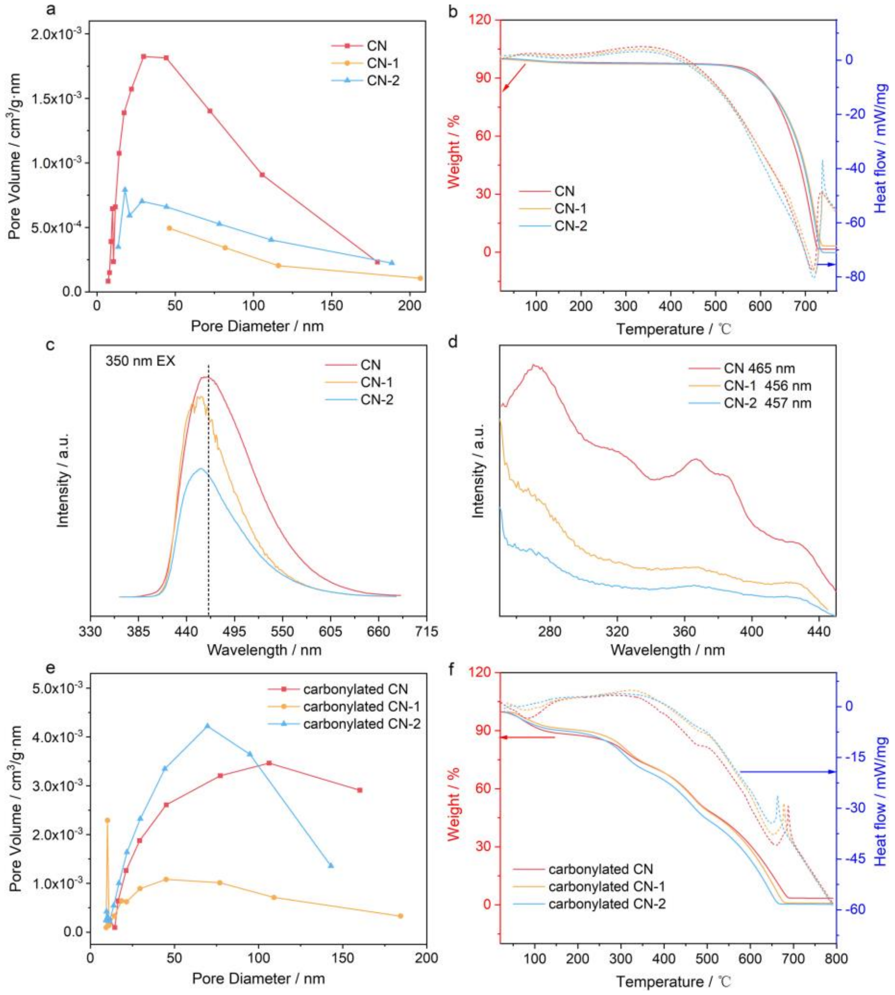

Figure S19. (a) Pore-size distribution analyses of CN, CN-1 and CN-2. (b) Thermogravimetric analyses (TGA) and differential scanning calorimetry (DSC) curves of CN, CN-1 and CN-2. (c) PL emission spectra of CN, CN-1 and CN-2. (d) PL excitation spectra monitored at the corresponding emission peaks of CN, CN-1 and CN-2. (e) Pore-size distribution analyses of carbonylated CN, carbonylated CN-1 and carbonylated CN-2. (e) TGA and DSC curves of carbonylated CN, carbonylated CN-1 and carbonylated CN-2.

However, the carbonylated CNs all exhibit excitation-energy dependent PL emissions (**Figure S20**), which excludes the effect of macroscopic inhomogeneities. Besides, detailed PL results of carbonylated CN-1 and carbonylated CN-2 including temperature-dependent PL spectra, excitation spectra, and time-resolved PL spectra gave similar key characteristics to those of carbonylated CN (see details in Additional Information 1). The above results not only exclude the impact of inhomogeneity on excitation-wavelength-dependent PL features of carbonylated CN, but also highlight the generality of carbonylation treatment in pursuing carbon-nitride-based anti-Kasha systems.

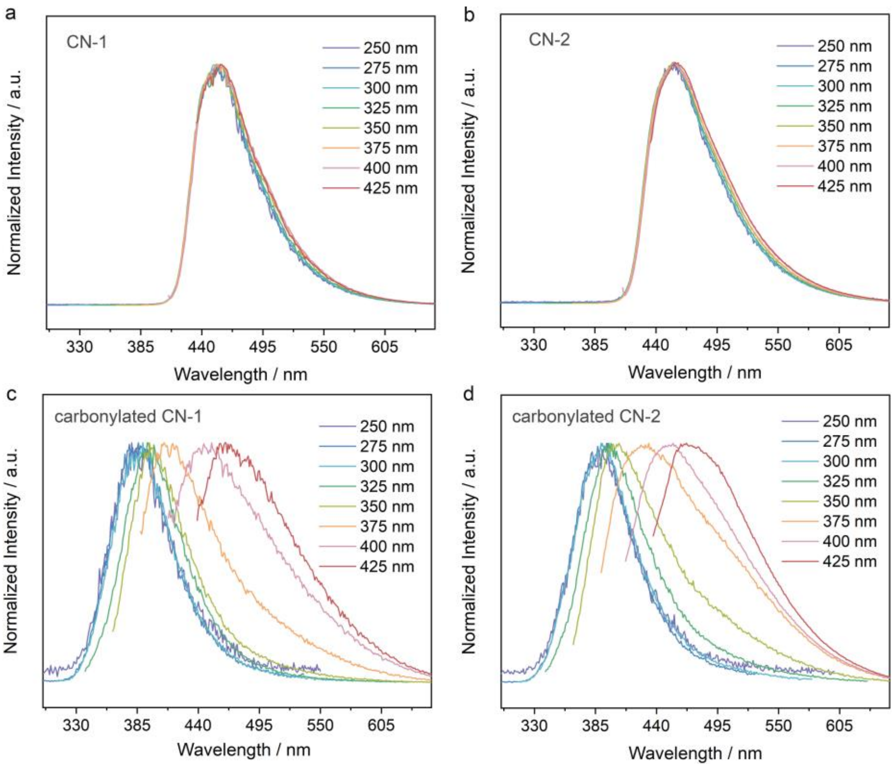

Figure S20. Normalized excitation-wavelength-dependent PL emission spectra of (a) CN-1, (b) CN-2, (c) carbonylated CN-1, and (d) carbonylated CN-2.

Additional Information 1. Details of the characterizations for control samples.

Structural characterizations

The X-ray diffraction (XRD) spectra of CN-1 and carbonylated CN-1 samples are shown in **Figure S21a**. CN-1 has two distinct peaks at 12.9 ° and 27.8 ° corresponding to the (100) and (002) diffraction planes.[3, 4] The similar XRD diffraction peaks of carbonylated CN-1 indicate that the layered structure of the carbon nitride is preserved. However, the shift and broadening of (002) peak for carbonylated CN-1 suggests its weakened periodicity induced by the oxidizing-acid treatment. Fourier transform infrared (FT-IR) spectroscopy showed the presence of oxygen-containing groups in carbonylated CN-1 (**Figure S21b**). As for CN-1 sample, characteristic absorption bands at ~810, 1200–1600, and 3000–3700 cm^{-1} were observed, presenting the breathing mode of triazine units, stretching vibration of heterocyclic ring, and N–H stretching, respectively.[5, 6] As for carbonylated CN-1, a new band at ~1720 cm^{-1} emerges, which can be ascribed to the stretching vibration of carbonyl groups.[7, 8] The information of functional groups was further interrogated by X-ray photoelectron spectroscopy (XPS). The intensity of O 1s spectrum for carbonylated CN-1 is much higher than that for CN-1 (**Figure S21c**), suggesting the promoted oxygen-containing species in the former. As indicated in the detailed peak analyses (**Figure S21d**), new peaks centered at 286.5, 287.8, and 289.0 eV emerge in carbonylated CN-1, corresponding to C–O, C=O, and –COOH, respectively.[7, 9] All of the above results demonstrated the successful synthesis of carbonylated CN-1 with rich carbonyl groups. **Figure S22** similarly demonstrated the successful synthesis of carbonylated CN-2.

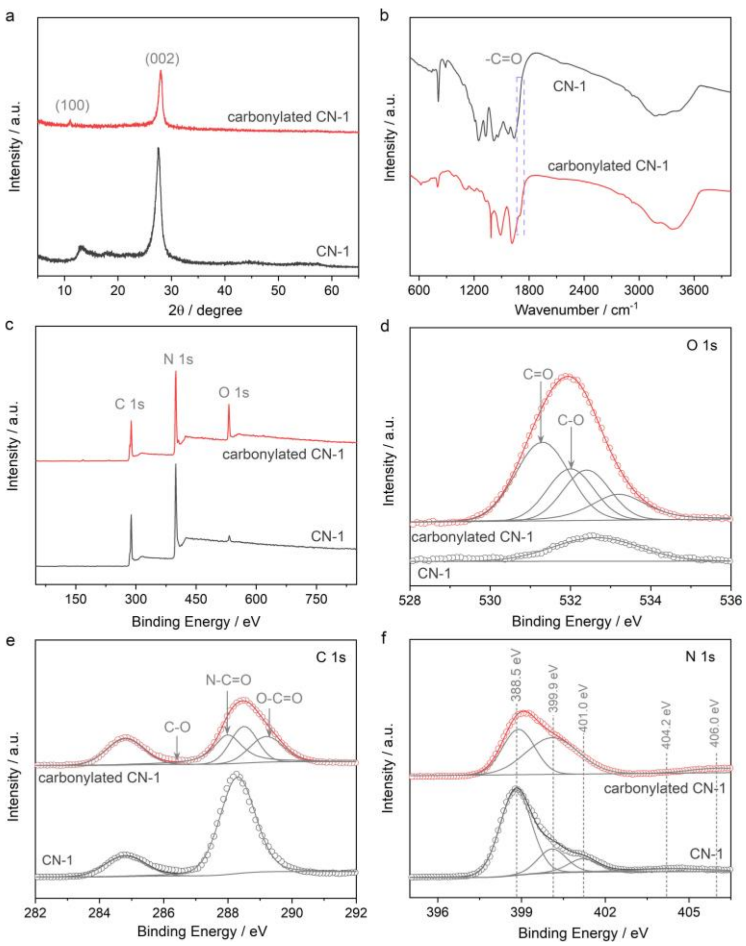

Figure S21. (a) XRD patterns, (b) FT-IR spectra, (c) XPS survey spectra, (d) O 1s spectra, (e) C 1s spectra and (f) N 1s spectra of CN-1 and carbonylated CN-1.

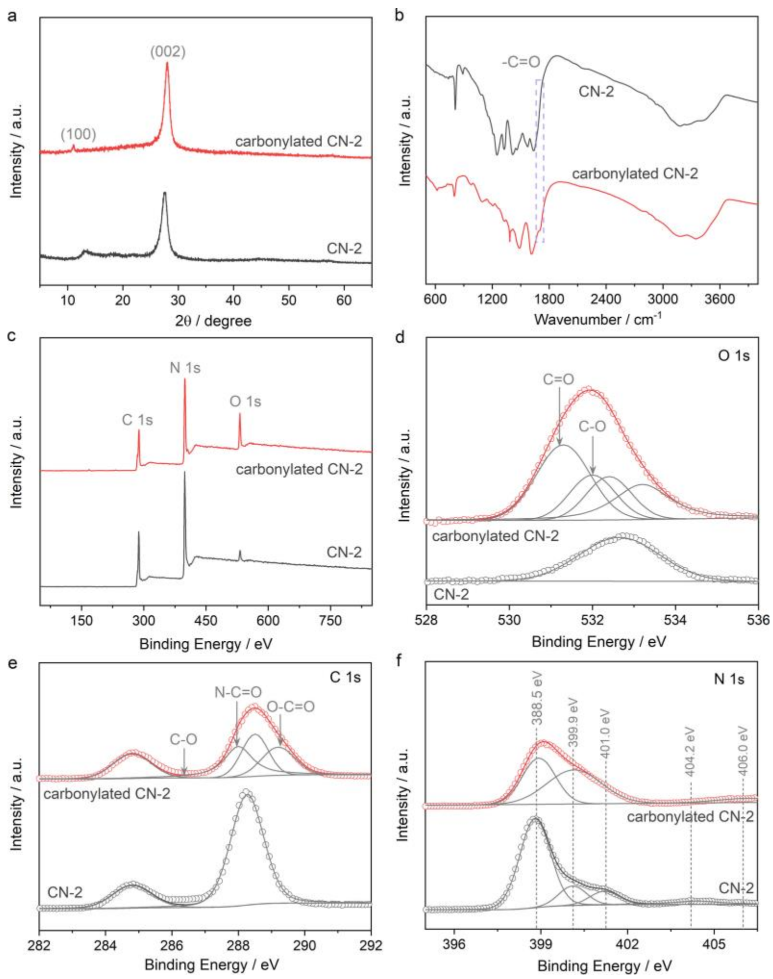

Figure S22. (a) XRD patterns, (b) FT-IR spectra, (c) XPS survey spectra, (d) O 1*s* spectra, (e) C 1*s* spectra and (f) N 1*s* spectra of CN-2 and carbonylated CN-2.

Photophysical characterizations

The photophysical properties of the materials were studied by UV–vis spectra and photoluminescence (PL) spectra. According to UV–vis spectra (**Figure S23**), a series of excitation wavelengths were selected from 250–425 nm with a 25-nm interval. As clearly seen from **Figure S24a**, the PL of CN-1 shows a typical Kasha behavior, where the emissions under different excitation wavelengths exhibit quite similar PL spectral line shapes. Such an excitation-energy-independent feature corresponds to the dominant emissions from the lowest excited state, arising as a result of the rapid cooling of photoinduced species. By contrast, the emissions of the carbonylated CN-1 sample were red-shifted with the increase of the excitation wavelengths (**Figure S24b**), which showed the excitation-energy-dependent phenomenon. It indicates the suppression of the rapid cooling process of high-energy excited state species in carbonylated CN-1. The PL excitation spectra (**Figure S24c** and **S24d**) show that the excitation spectra of both pristine and carbonylated CN-1 at different monitoring wavelengths exhibit several general bands around 272, 365, and 423 nm, suggesting that all the emission originated from the intrinsically excited state of the carbonylated CN-1 rather than the impurity- or non-homogeneous-induced state. In addition, time-resolved PL measurements were also performed, and as shown in **Figure S24e**, the time-resolved PL spectra of the CN-1 samples under 350 and 375 nm excitation exhibited very similar decay curves with almost the same average PL lifetimes (i.e., ~6.22 and 6.33 ns), besides, the statistical weights of the decay components under 350- and 375-nm excitations are also quite close (**Table S2**). In contrast, the decay kinetics of carbonylated CN-1 at different excitation wavelengths showed significant differences (**Figure S24f**), with average PL lifetimes of ~0.28 and 3.91 ns under 350 nm and 375 nm excitation, respectively. And decay-component statistical weights under 350- and 375-nm excitations of carbonylated CN-1 are distinct (**Table S2**). The above results demonstrated that the carbonylated CN-1 also exhibits excitation-energy-dependent photophysical properties.

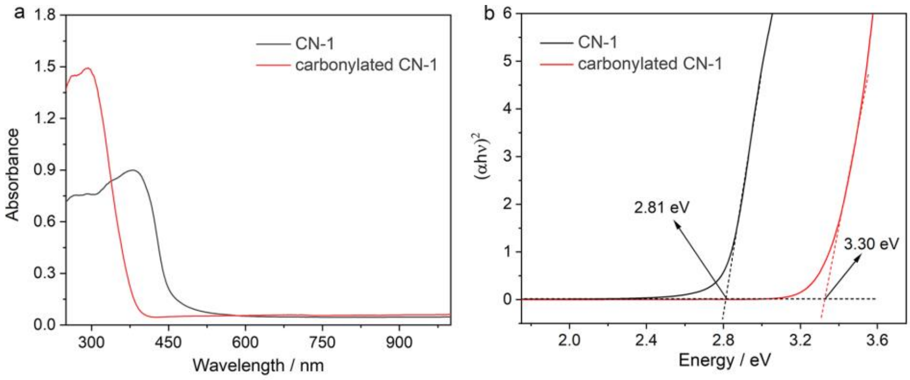

Figure S23. (a) UV–vis spectra and (b) the corresponding Tauc plots of CN-1 and carbonylated CN-1.

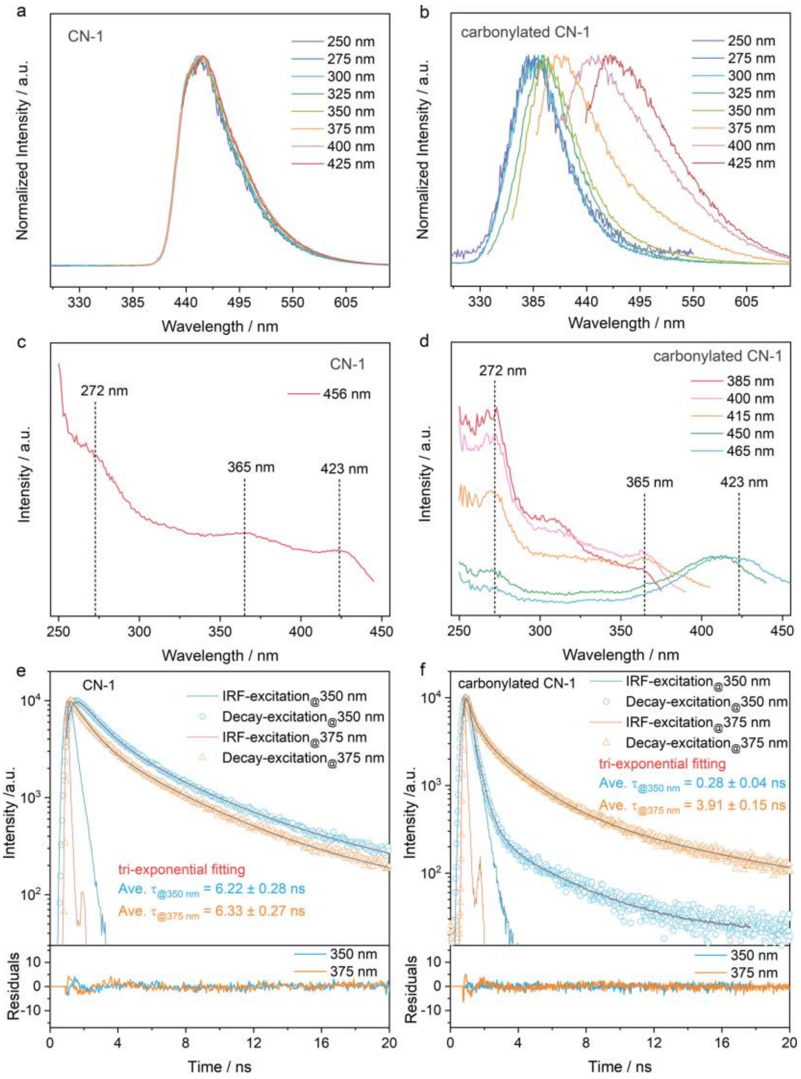

Figure S24. Normalized PL emission spectra of (a) CN-1 and (b) carbonylated CN-1 under different excitation wavelengths. PL excitation spectra monitored at the

corresponding emission peaks of (c) CN-1 and (d) carbonylated CN-1. Time-resolved PL spectra under 350- and 375- nm excitation (monitored at corresponding emission peaks) of (e) CN-1 and (f) carbonylated CN-1.

Table S2. Tri-exponential fitting results of time-resolved PL spectra of CN-1 and carbonylated CN-1.

Materials	CN-1		carbonylated CN-1	
Excitation wavelength	350 nm	375 nm	350 nm	375 nm
T1 / ns	4.15 ±0.25 (51%)	4.09 ±0.23 (53%)	0.71 ±0.14 (4%)	2.08 ±0.11 (49%)
T2 / ns	16.56 ±0.62 (23%)	17.69 ±0.61 (22%)	3.78 ±0.30 (6%)	8.80 ±0.28 (32%)
T3 / ns	1.14 ±0.04 (26%)	1.01 ±0.03 (25%)	0.02 ±0.01 (90%)	0.40 ±0.02 (19%)
Average LifeTime / ns	6.22 ±0.28	6.33 ±0.27	0.28 ±0.04	3.91 ±0.15

Similarly, the same excitation-energy-dependent photophysical behavior is observed in carbonylated CN-2 (**Figure S25**, **Figure S26**, **Table S3**).

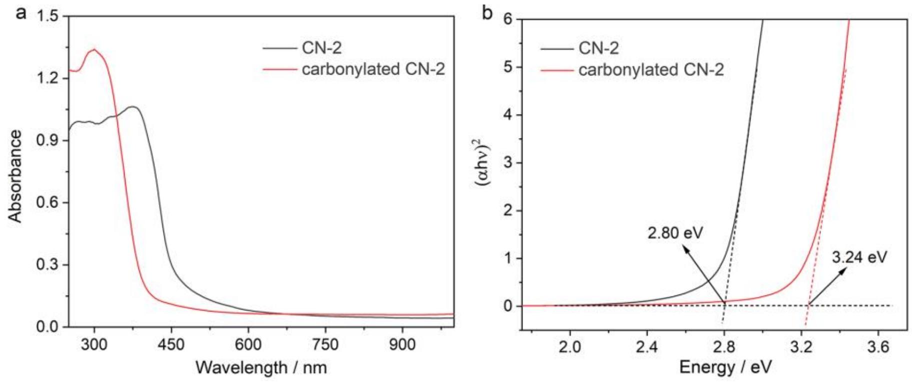

Figure S25. (a) UV–vis spectra and (b) the corresponding Tauc plots of CN-2 and carbonylated CN-2.

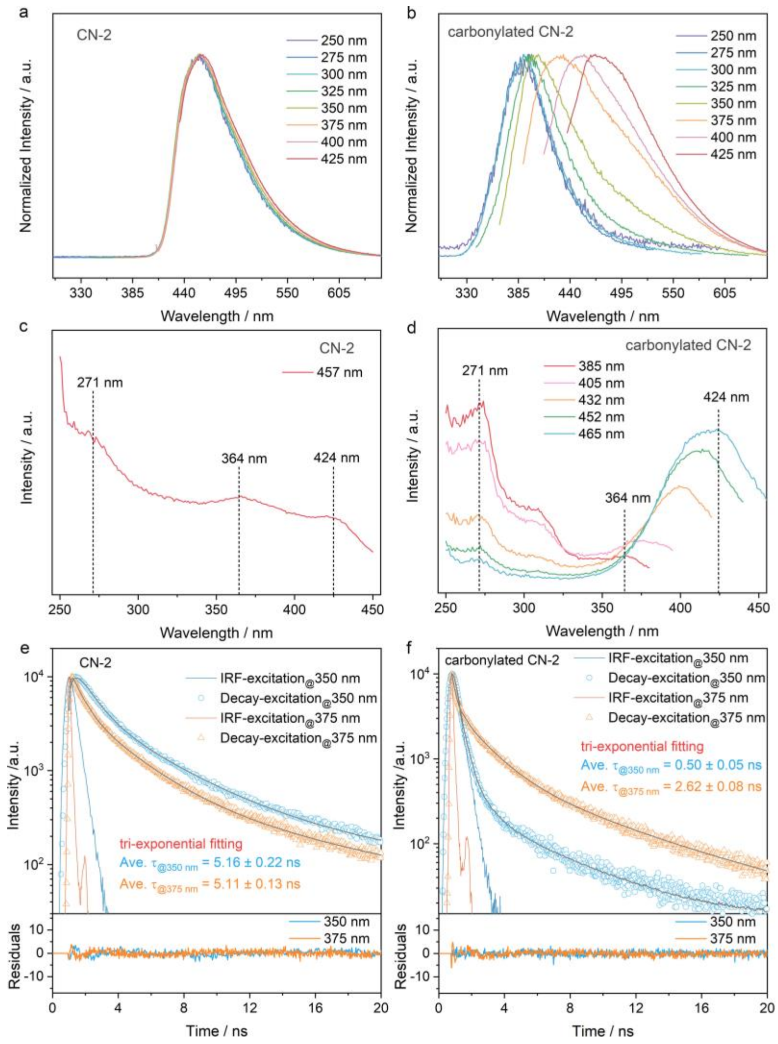

Figure S26. Normalized PL emission spectra of (a) CN-2 and (b) carbonylated CN-2 under different excitation wavelengths. PL excitation spectra monitored at the corresponding emission peaks of (c) CN-2 and (d) carbonylated CN-2. Time-resolved PL spectra under 350- and 375- nm excitation (monitored at corresponding emission peaks) of (e) CN-2 and (f) carbonylated CN-2.

Table S3. Tri-exponential fitting results of time-resolved PL spectra of CN-2 and carbonylated CN-2.

Materials	CN-2		carbonylated CN-2	
Excitation wavelength	350 nm	375 nm	350 nm	375 nm
T1 / ns	3.57 ±0.21 (49%)	3.39 ±0.13 (51%)	0.91 ±0.18 (7%)	1.56 ±0.08 (39%)
T2 / ns	15.01 ±0.52 (21%)	15.23 ±0.27 (20%)	4.42 ±0.31 (9%)	6.05 ±0.15 (32%)
T3 / ns	0.92 ±0.03 (30%)	0.91 ±0.02 (29%)	0.04 ±0.01 (84%)	0.22 ±0.01 (29%)
Average LifeTime / ns	5.16 ±0.22	5.11 ±0.13	0.50 ±0.05	2.62 ±0.08

Figure S27 shows the temperature-dependent PL spectrum of carbonylated CN-1. Significant splitting peaks can be seen at 30 K. At progressively higher temperatures, splitting peaks broaden and begin to overlap, and broad featureless peaks are observed at 298 K. This is related to the inhibition of electron-vibration coupling at low temperatures.[10, 11] Meanwhile, as can be seen from **Figure S27a**, the emission peaks at different excitation wavelengths are composed of some specific bands like 391-, 408-, 430-, 456- nm. The features could be interpreted as that the whole emissions consist of a series of emissions from specific excited states, and excitation energy mainly impact the proportions of these emissions. And the same properties of carbonylated CN-2 are also demonstrated in **Figure S28**.

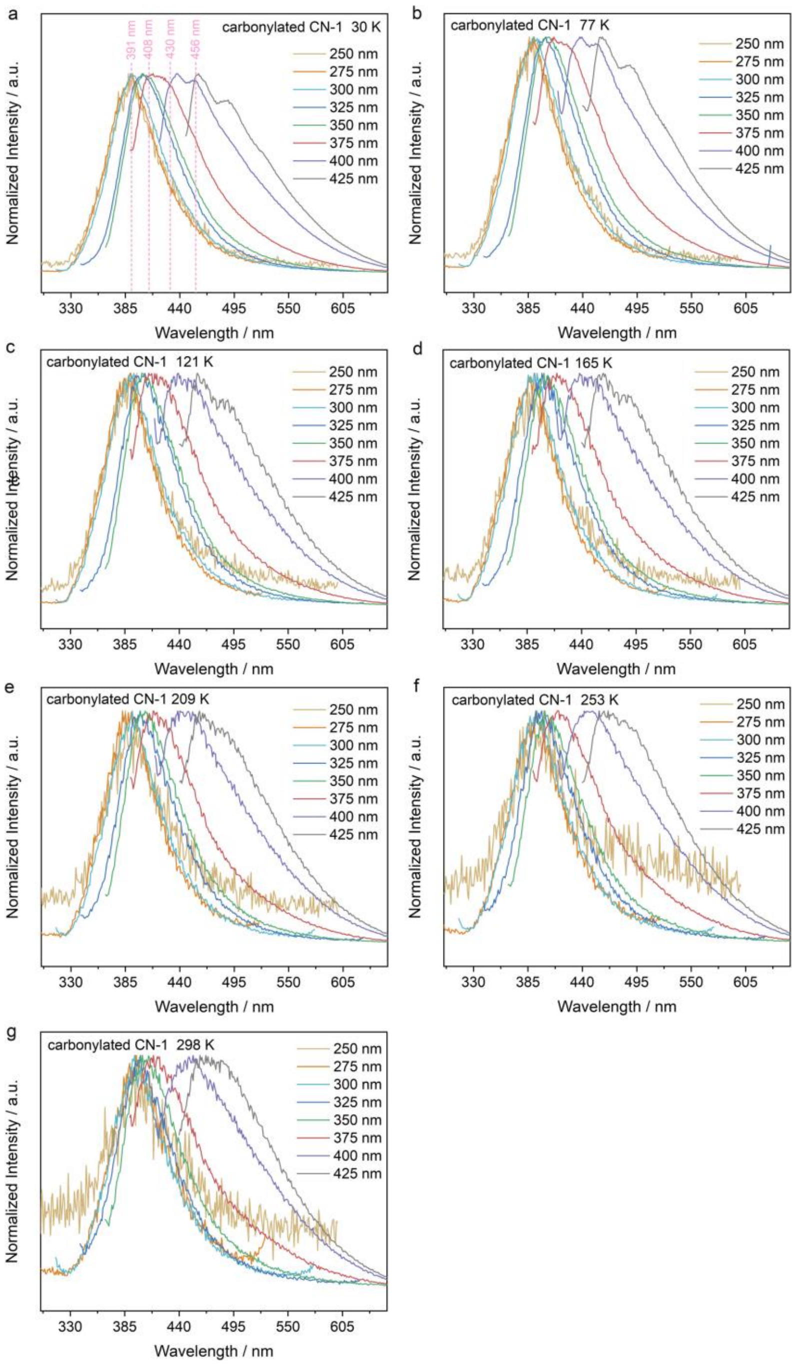

Figure S27. Normalized PL emission spectra of carbonylated CN-1 under different excitation wavelengths at (a) 30 K, (b) 77 K, (c) 121 K, (d) 165 K, (e) 209 K, (f) 253 K, (g) 298 K.

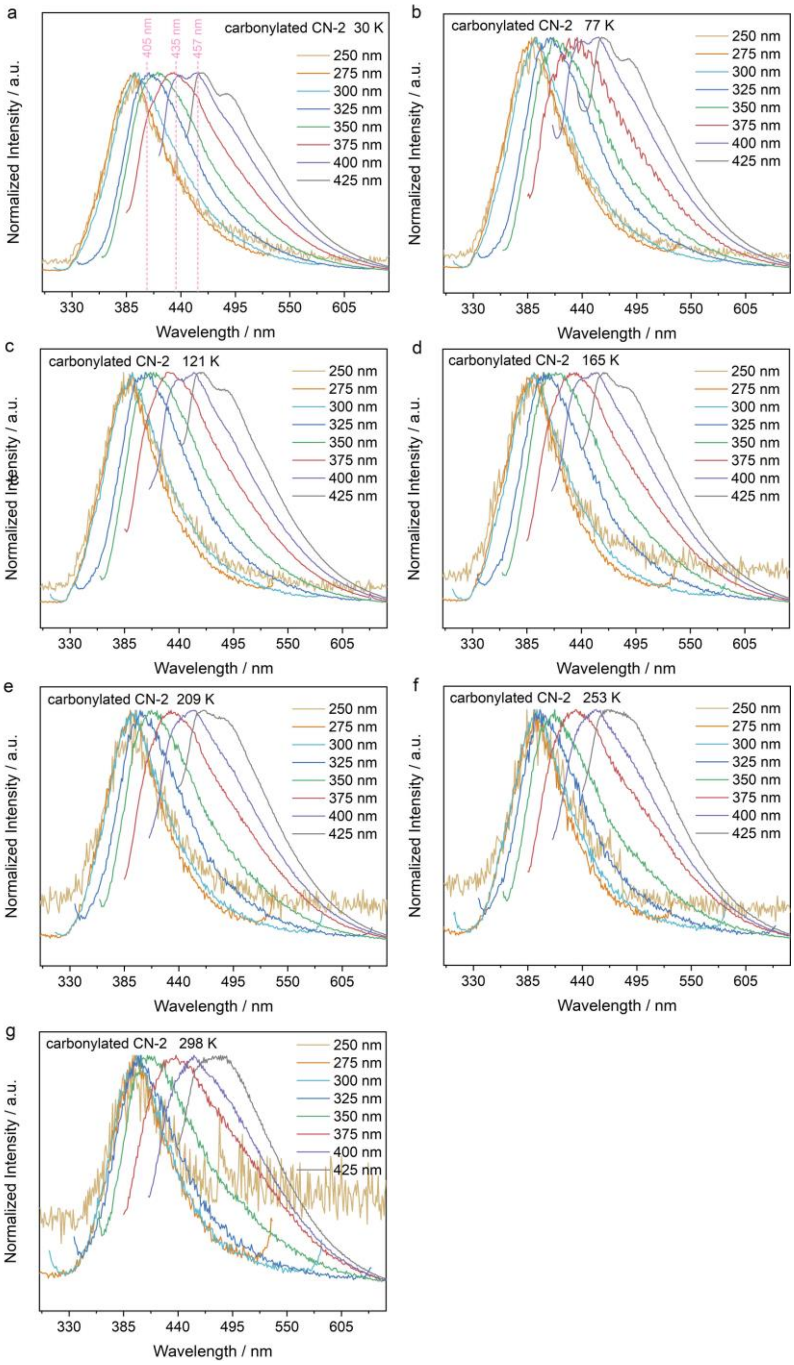

Figure S28. Normalized PL emission spectra of carbonylated CN-2 under different excitation wavelengths at (a) 30 K, (b) 77 K, (c) 121 K, (d) 165 K, (e) 209 K, (f) 253 K, (g) 298 K.

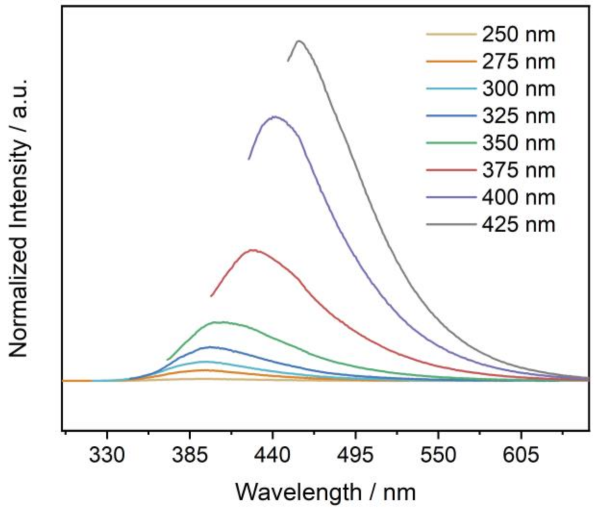

Figure S29. PL emission spectra of carbonylated CN under different excitation wavelengths.

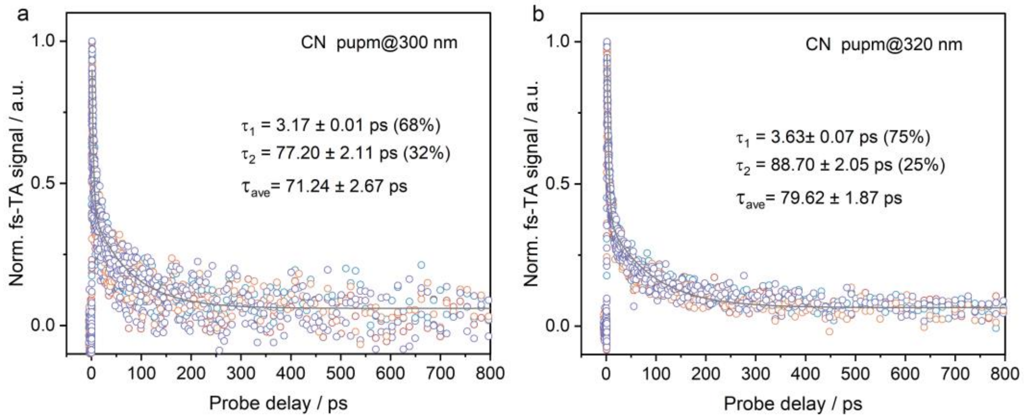

Figure S30. The fs-TA kinetic traces excited at (c) 300 nm and (d) 320 nm of CN.

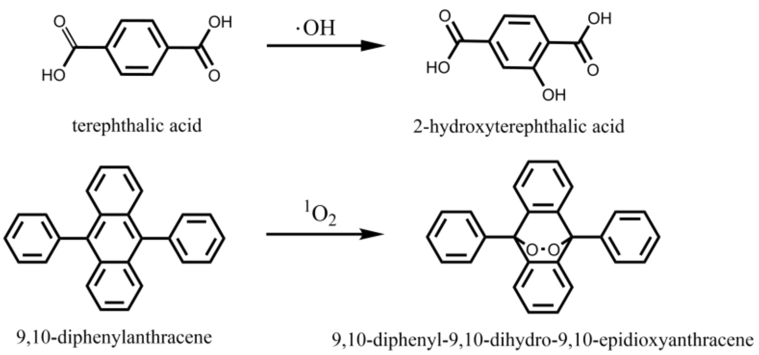

Figure S31. Schematic diagram of the •OH and $^1O_2$ detections. The trappings of •OH and $^1O_2$ by terephthalic acid and 9,10-diphenylanthracene lead to formation of 2-hydroxy-p-benzenedicarboxylic acid and 9,10-diphenylanthracene endoperoxide, respectively.

Note S5: Reactive oxygen generation by reduced-carbonylated CN.

TA and DPA tests were carried out on reduced-carbonylated CN to compare the photoluminescence of the terephthalic acid solution and the UV–vis absorption intensity of the DPA solution under illuminations at 320 nm and 380 nm. This trend of reverse •OH and $^1O_2$ generation turned to be inconspicuous, demonstrating that the reverse -•OH and $^1O_2$ production of carbonylated CN under 320 and 380 nm light is closely related to the carbonyl group.

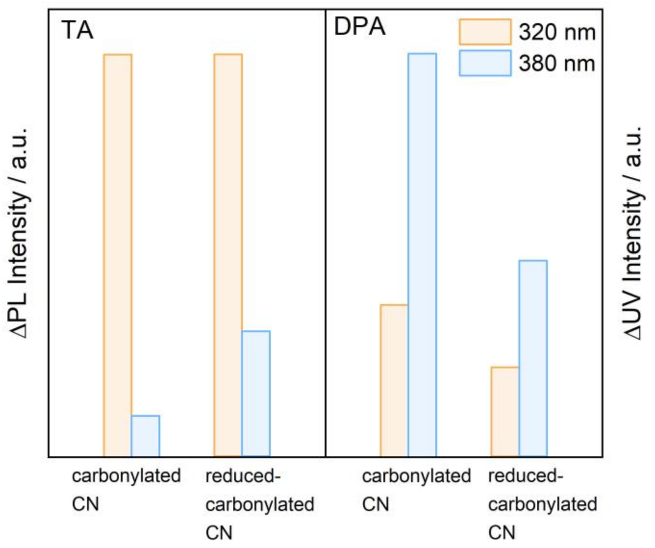

Figure S32. The PL and UV intensity increments of terephthalic acid (TA) (within 60 min) and DPA solutions (within 15 min) at 320 nm/380 nm for carbonylated CN and reduced-carbonylated CN.

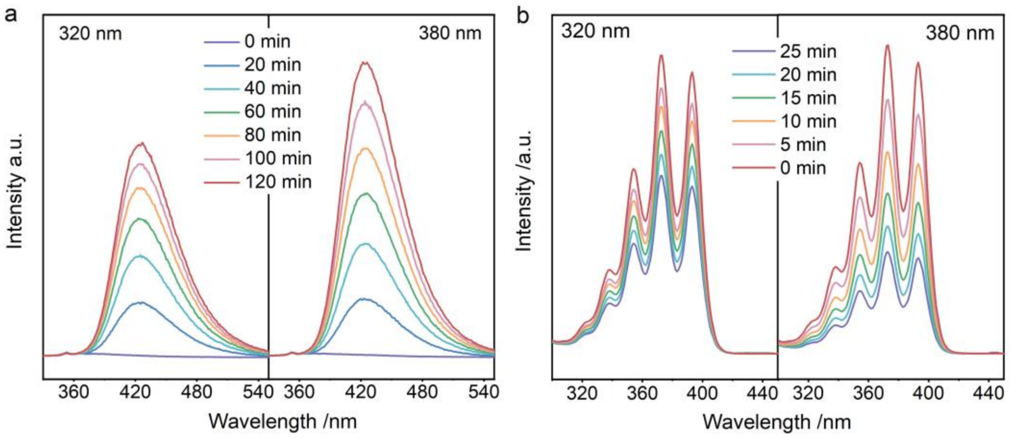

Figure S33. (a) Terephthalic acid measurements for •OH detection (b) DPA measurements for $^1O_2$ detection at 320 nm and 380 nm for CN.

Note S6: Structural characterization and PL testing of carbonylated CN samples before and after photoreaction.

We conducted structural characterization and PL tests on the carbonylated CN samples after the photoreaction (including the terephthalic acid reaction and the DPA reaction). The results are shown in **Figures S34-S37**. After a 2-hour catalytic reaction, the TEM images (**Figure S34**) distinctly reveal the preservation of the layered stacking structure. The XRD pattern (**Figure S35a**) exhibits no notable alterations in the position or shape of the diffraction peaks. The infrared spectrum (**Figure S35b**) confirms that the C-N conjugated framework of the carbonylated CN remains substantially intact, with the carbonyl group still evident. XPS analysis (**Figure S36**) further indicates that the functional groups' types and relative concentrations are largely unchanged before and after the photoreaction. Consequently, the carbonylated CN demonstrates remarkable structural stability post-photoreaction. Significantly, the PL spectrum (**Figure S37**) illustrates that the distinctive excitation-energy-dependent characteristic of the carbonylated CN is consistently maintained throughout the photoreaction process.

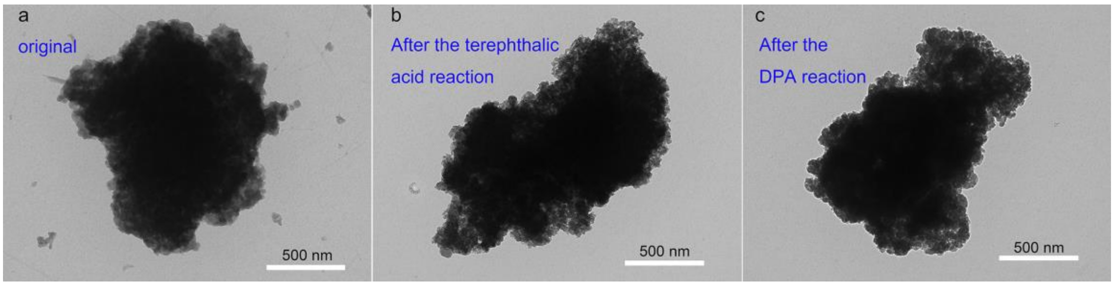

Figure S34. TEM images of carbonylated CN, (a) original and after (b) the terephthalic acid reaction and (c) the DPA reaction.

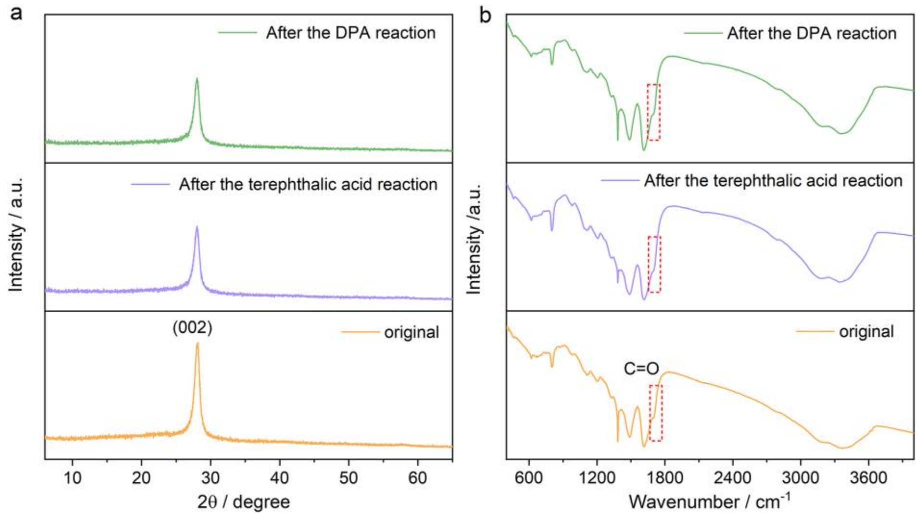

Figure S35. (a) XRD patterns, (b) FT-IR spectra of original carbonylated CN and after the terephthalic acid reaction and the DPA reaction.

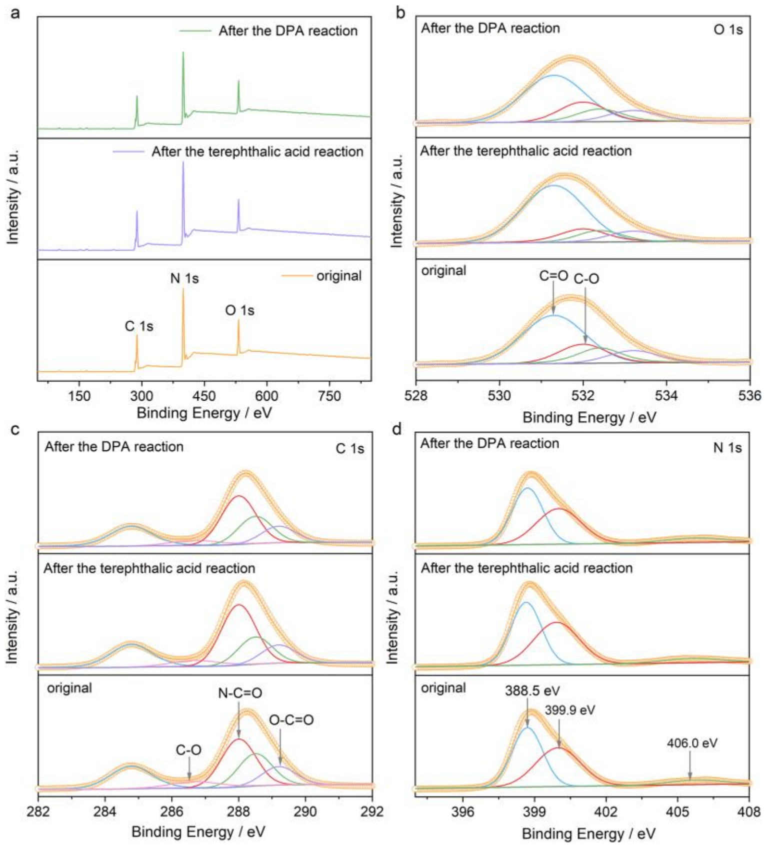

Figure S36. (a) XPS survey spectra, (b) O 1*s* spectra, (c) C 1*s* spectra, (d) N 1*s* spectra of original carbonylated CN and after the terephthalic acid reaction and the DPA reaction.

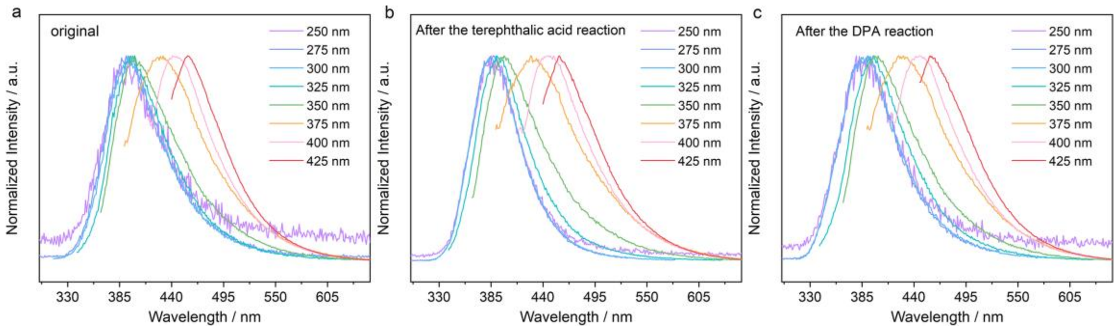

Figure S37. Normalized PL emission spectra of carbonylated CN, (a) original and after (b) the terephthalic acid reaction and (c) the DPA reaction.

Note S7: The isotope-labeling experiment.

In this study, we propose that singlet oxygen ($^1O_2$) is generated via an exciton energy transfer process, where excitons created by carbonylated CN convey energy to oxygen molecules, exciting them to $^1O_2$. Hydroxyl radicals (•OH) are produced through a charge carrier-mediated process, with holes generated from the photoexcitation of carbonylated CN directly oxidizing water molecules to form •OH. To ascertain the origins of $^1O_2$ and •OH generated in the photoreaction, we performed isotope-labeling experiments using $^{18}O_2$ and $H_2{}^{18}O$ as the corresponding substrates. Terephthalic acid and 9,10-diphenylanthracene served as molecular probes to detect the production of photo-catalyzed •OH and $^1O_2$, respectively. Upon reaction in $H_2{}^{16}O$, only 2-hydroxyterephthalic acid/^{16}O was identified in the terephthalic acid solution post-photoreaction (**Figure S38a**). In contrast, when utilizing ^{18}O-labeled H_2O, 2-hydroxyterephthalic acid/^{18}O was detected in the solution (**Figure S38b**), with minor ^{16}O-related product signals potentially arising from the conversion of adsorbed water on the catalyst's surface. This confirms that photoreaction-generated •OH stems from the oxidation of H_2O. Similarly, when reacting in $^{16}O_2$, only 9,10-diphenylanthracene endoperoxide/^{16}O was detected in the 9,10-diphenylanthracene solution after the photoreaction (**Figure S39a**). However, with ^{18}O-labeled O_2, 9,10-diphenylanthracene endoperoxide/^{18}O was present in the solution (**Figure S39b**), with minor ^{16}O-related product signals possibly resulting from the presence of $^{16}O_2$ in the $^{18}O_2$ mixture. This confirms that the photoreaction-generated $^1O_2$ originates from the conversion of O_2.

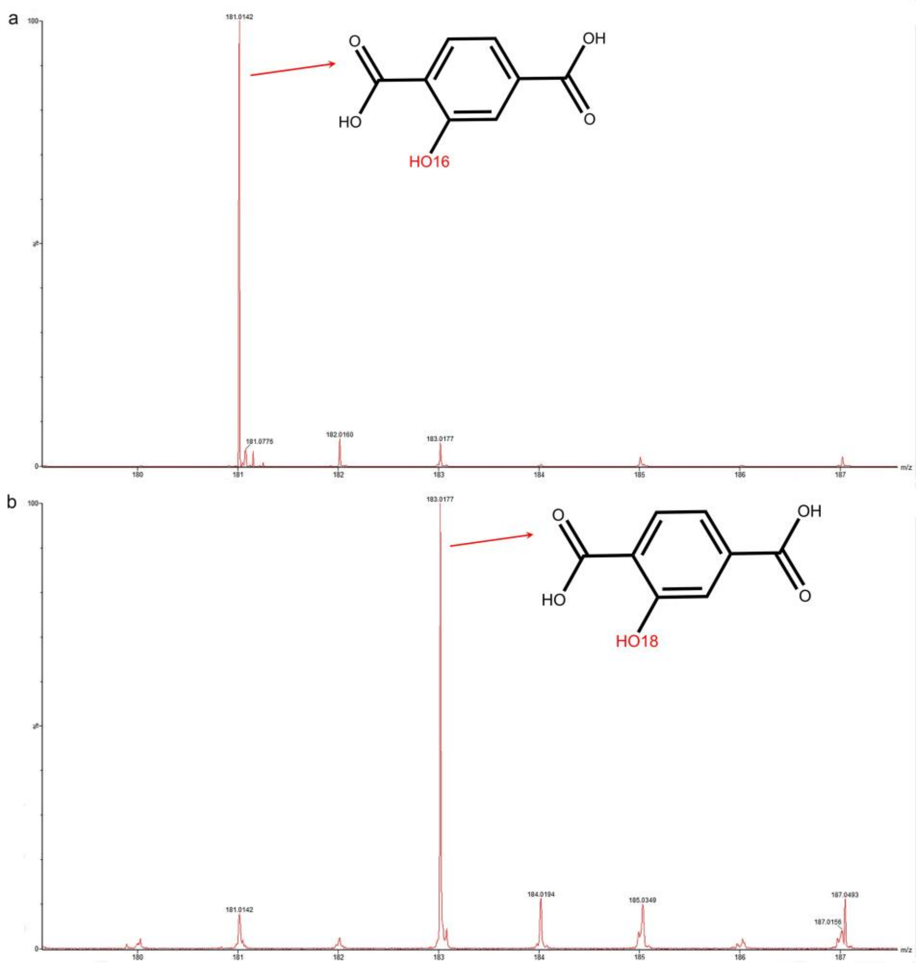

Figure S38. Mass spectra of the products of TA measurements in presence of (a) $H_2{}^{16}O$ and (b) $H_2{}^{18}O$. The mass-to-charge ratios (m/z) of 2-hydroxyterephthalic acid/^{16}O and 2-hydroxyterephthalic acid/^{18}O are 181.0142 and 183.0177, respectively.

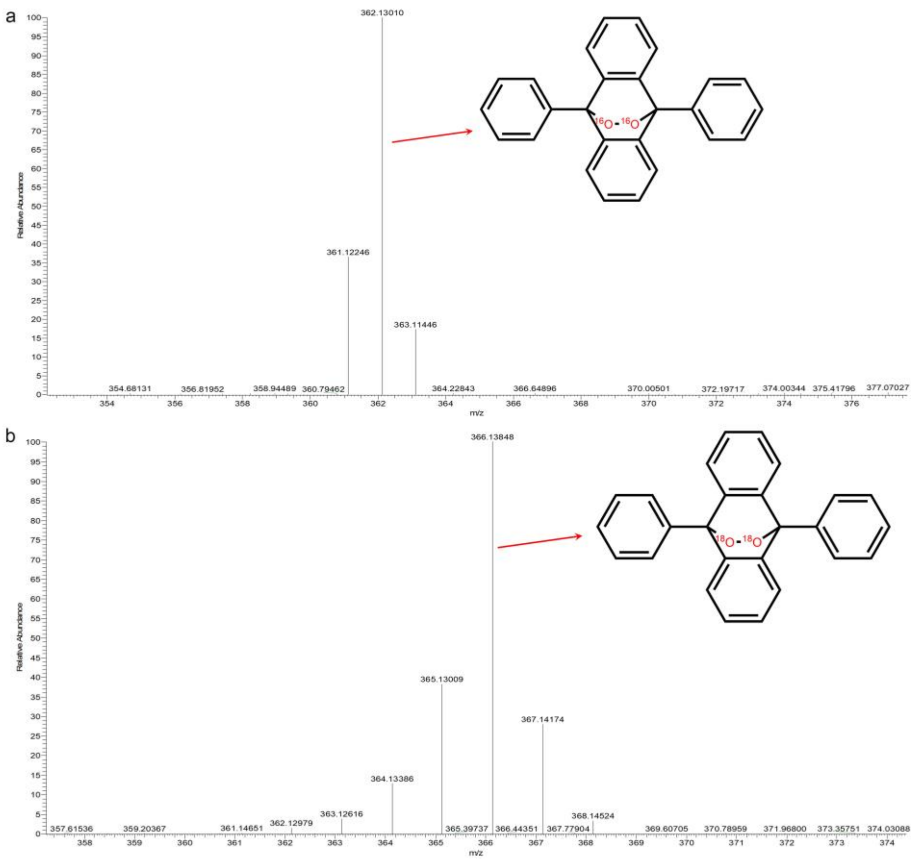

Figure S39. Mass spectra of 9,10-diphenylanthracene endoperoxide reacting in (a) $^{16}O_2$ (b) $^{18}O_2$. The mass-to-charge ratios (m/z) of 9,10-diphenylanthracene endoperoxide/^{16}O and 9,10-diphenylanthracene endoperoxide/^{18}O are 362.13 and 366.14, respectively.

Note S8: The electronic band structure analyses.

We determined the bandgaps of CN and carbonylated CN to be 2.75 eV and 3.06 eV, respectively, through UV-vis absorption spectra (**Figure S6a, b**). We then performed Mott-Schottky measurements on CN and carbonylated CN, finding their flat-band potentials to be −0.74 eV and −0.69 eV (vs. Ag/AgCl) (**Figure S40a**). From this, we can calculate the conduction-band minima of CN and carbonylated CN to be −0.39 eV and −0.44 eV (vs. NHE). Based on the bandgaps, we ascertained the valence-band maxima of CN and carbonylated CN to be 2.36 eV and 2.62 eV (vs. NHE), respectively. Consequently, we were able to construct the electronic band structure as depicted in **Figure S40b**. The valence-band maximum of CN is marginally lower than the redox potential of H_2O/•OH (2.38 V, vs. NHE); nonetheless, the adsorption of water molecules on the CN surface can lower the energy barrier, facilitating the oxidation of water to •OH. The valence-band maximum of carbonylated CN is above the redox potential of H_2O/•OH, enabling direct oxidation of water to produce •OH via photoinduced holes in the valence band.

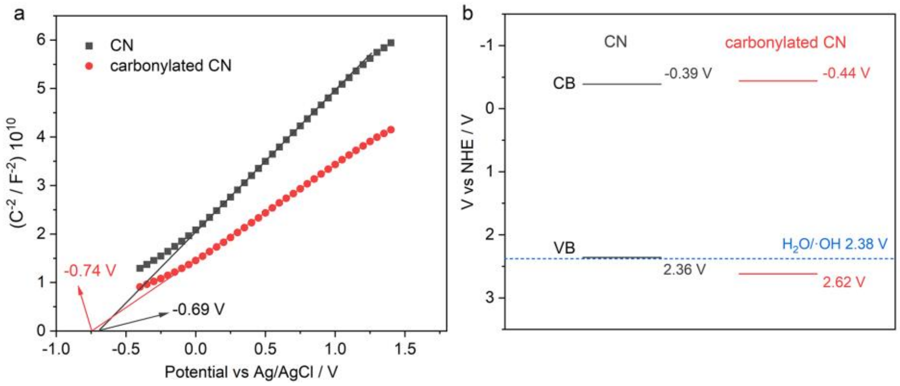

Figure S40. (c) Mott-Schottky curves and (d) energy level diagrams of CN and carbonylated CN.

Note S9: Exploring the trend of ROS generation in carbonylated CN and CN.

The Saha–Langmuir equation has been widely used to describe the ionization of excitons in thermal equilibrium. The basic form of the Saha–Langmuir equation is displayed as

$$\frac{x^2}{1-x} = \frac{1}{n}\left(\frac{2\pi\mu k_B T}{h^2}\right)^{3/2} e^{-\frac{E_B}{k_B T}}$$

where x and $1 - x$ are relative ratios of free carriers (electron or hole) and exciton in total excitation density, n is the sum of excitons and free carriers, μ is the reduced effective mass, k_B, T, and h are the Boltzmann constant, temperature, and Planck's constant, respectively, and E_B is the exciton binding energy.[12]

It should be emphasized that T should be exciton temperature, rather than ambient or lattice temperature,[13, 14] which seems to have been confused in many reports. In general, such a confusion does not lead to large deviations, owing to the fact that rapid hot-exciton cooling results in the fast equilibrium between exciton and ambient temperatures. However, as for anti-Kasha systems, the scenario could be quite different: owing to the blocking of hot-exciton cooling, excitons tend to be accommodated at high-energy excited states, which process higher exciton temperature than those accommodated at the lowest excited states. As for carbonylated CN, higher energy excitation leads to the accumulation of excitons with higher (exciton) temperature, thereby bring the thermal equilibrium between excitons and free carriers to the latter. This feature could be related the unique tendencies of hydroxyl radical (carrier-related mechanism) and singlet oxygen (exciton-related mechanism) generation. Note that hot excitons in CN system also possess higher temperature that could facilitate their dissociation, whereas the fast cooling (sub-picosecond level) is superior to exciton dissociation (picosecond level). In fact, the facile dissociation of hot or higher-lying excitons generated under high-energy excitation has been widely observed in polymeric systems, where the excess energy (to some extent, related to exciton temperature) is crucial to the following dissociation.[15-17]

As for carbonylated CN, compared with the scenario in low-energy excited states, the equilibrium between charge carriers and excitons in high-energy excited states would shift toward the charge-carrier side, thereby resulting in a promoted charge-carrier concentration and a reduced exciton concentration (as shown in Figure 4e). This phenomenon could be rationalized as follows[18]: hot excitons generated under high-energy excitation would quickly relax their excess energies via exciton–phonon scattering; the exchange of thermal energy between electronic and phonon subsystems would lead to promoted effective temperature (in detail, lattice temperature) over the conjugation segments of polymer chains; the elevated temperature favors the exciton dissociation by boosting charge transfer states into free carriers or polarons.

Note S10: Transient photocurrent response testing of carbonylated CN and CN.

The photocurrent response of carbonylated CN and CN was tested at 320, 350, and 380 nm. The instantaneous photocurrent of carbonylated CN at 380 nm was extremely low, representing a very low carrier concentration. With the increase of excitation energy, the instantaneous photocurrent increases dramatically, illustrating the substantial increase of carrier concentration. The balance between carriers and excitons is demonstrated to be shifted to the carrier side for high-energy excitation compared with low-energy excitation. As for the pristine CN, its instantaneous photocurrent is almost indistinguishable under 320 and 350 nm excitation, while the slightly reduced instantaneous photocurrent under 380 nm excitation is related to its reduced light absorption efficiency.

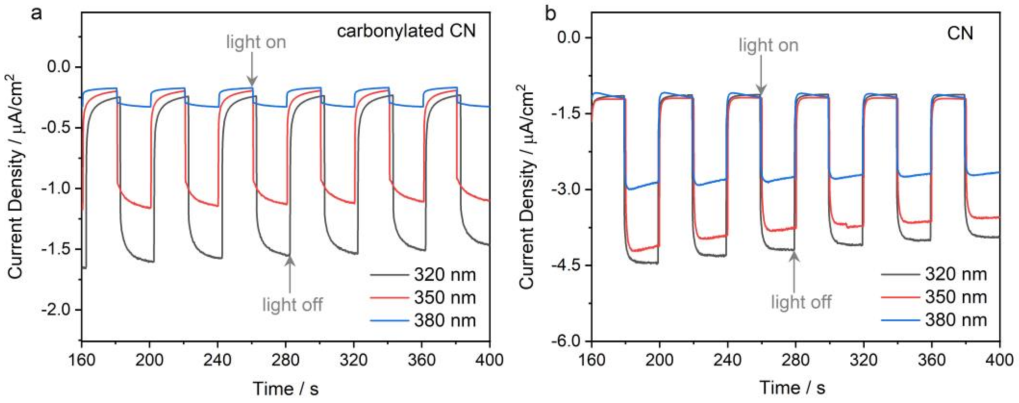

Figure S41. Periodic on/off photocurrent responses of (a) carbonylated CN and (b) CN.

Note S11: Excitation-density-dependent ROS generation test for carbonylated CN.

We further carried out excitation-density-dependent ROS generation tests for carbonylated CN sample. The excitation wavelength was chosen at 320 nm, and the optical power density was controlled from 5 to 30 mW (with a 5-mW interval). As shown in **Figure S41**, DPA- and terephthalic-acid-based tests confirm that both •OH and $^1O_2$ productions increase with optical power density. However, when the optical power density is increased from 5 mW to 30 mW, •OH generation increases by a factor of 5, while $^1O_2$ generation increases by a factor of ~ 2.5. The more favorable •OH generation under higher excitation density due to higher exciton ratio under higher excitation density. This is because the higher power density means that more thermal excitons will release energy into the lattice, contributing further to exciton dissociation.

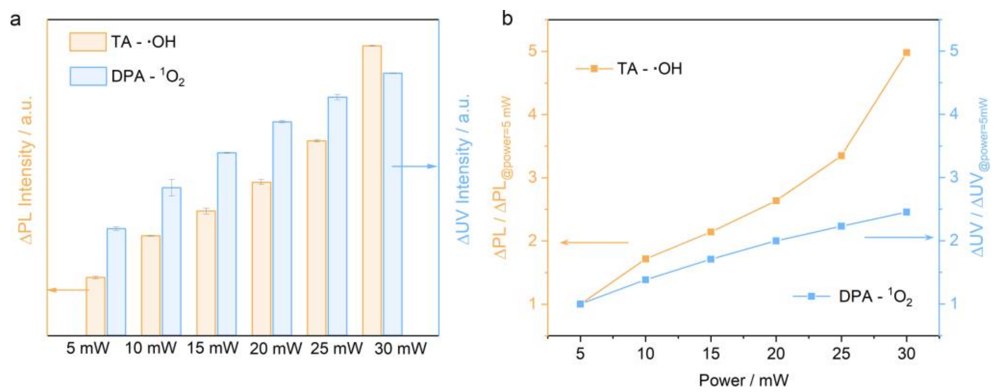

Figure S42. PL and UV intensity (a) increments and (b) ratios of carbonylated CN in terephthalic acid (TA) solution (within 120 min) and DPA solution (within 30 min) at 320 nm unedr different power densities.

Reference

(1) Liu, Y.; Deng, R.; Wang, Z.; Liu, H. Carboxyl-functionalized graphene oxide–polyaniline composite as a promising supercapacitor material. *J. Mater. Chem.* **2012**, *22*, 13619-13624.

(2) Liang, J.; Jing, C.; Wang, J.; Men, Y. Photocatalytic Reduction of Cr (VI) over g-C_3N_4 Photocatalysts Synthesized by Different Precursors. *Molecules* **2021**, *26*, 7054.

(3) Cao, S.; Low, J.; Yu, J.; Jaroniec, M. Polymeric Photocatalysts Based on Graphitic Carbon Nitride. *Adv. Mater.* **2015**, *27*, 2150-2176.

(4) Liu, Y.-N.; Shen, C.-C.; Jiang, N.; Zhao, Z.-W.; Zhou, X.; Zhao, S.-J.; Xu, A.-W. g-C_3N_4 Hydrogen-Bonding Viologen for Significantly Enhanced Visible-Light Photocatalytic H2 Evolution. *ACS Catal.* **2017**, *7*, 8228-8234.

(5) Wang, W.; Zhang, H.; Zhang, S.; Liu, Y.; Wang, G.; Sun, C.; Zhao, H. Potassium-Ion-Assisted Regeneration of Active Cyano Groups in Carbon Nitride Nanoribbons: Visible-Light-Driven Photocatalytic Nitrogen Reduction. *Angew. Chem. Int. Ed.* **2019**, *58*, 16644-16650.

(6) Wang, N.; Wang, D.; Wu, A.; Wang, S.; Li, Z.; Jin, C.; Dong, Y.; Kong, F.; Tian, C.; Fu, H. Few-layered MoS_2 anchored on 2D porous C_3N_4 nanosheets for Pt-free photocatalytic hydrogen evolution. *Nano Res.* **2023**, *16*, 3524-3535.

(7) Wang, H.; Jiang, S.; Chen, S.; Li, D.; Zhang, X.; Shao, W.; Sun, X.; Xie, J.; Zhao, Z.; Zhang, Q.; et al. Enhanced Singlet Oxygen Generation in Oxidized Graphitic Carbon Nitride for Organic Synthesis. *Adv. Mater.* **2016**, *28*, 6940-6945.

(8) Yeh, T.-F.; Syu, J.-M.; Cheng, C.; Chang, T.-H.; Teng, H. Graphite Oxide as a Photocatalyst for Hydrogen Production from Water. *Adv. Funct. Mater.* **2010**, *20*, 2255-2262.

(9) Compton, O. C.; Dikin, D. A.; Putz, K. W.; Brinson, L. C.; Nguyen, S. T. Electrically Conductive "Alkylated" Graphene Paper via Chemical Reduction of Amine-Functionalized Graphene Oxide Paper. *Adv. Mater.* **2010**, *22*, 892-896.

(10) Parveen, S.; Paul, K. K.; Das, R.; Giri, P. K. Large exciton binding energy, high photoluminescence quantum yield and improved photostability of organo-metal halide hybrid perovskite quantum dots grown on a mesoporous titanium dioxide template. *J. Colloid Interface Sci.* **2019**, *539*, 619-633.

(11) Yu, P.; Wen, X.; Toh, Y.-R.; Tang, J. Temperature-Dependent Fluorescence in Carbon Dots. *J. Phys.*

Chem. C **2012**, *116*, 25552-25557.

(12) D'Innocenzo, V.; Grancini, G.; Alcocer, M. J. P.; Kandada, A. R. S.; Stranks, S. D.; Lee, M. M.; Lanzani, G.; Snaith, H. J.; Petrozza, A. Excitons versus free charges in organo-lead tri-halide perovskites. *Nat. Commun.* **2014**, *5*, 3586.

(13) Beck, M.; Hübner, J.; Oestreich, M.; Bieker, S.; Henn, T.; Kiessling, T.; Ossau, W.; Molenkamp, L. W. Thermodynamic origin of the slow free exciton photoluminescence rise in GaAs. *Physical Review B* **2016**, *93*, 081204.

(14) Bieker, S.; Henn, T.; Kiessling, T.; Ossau, W.; Molenkamp, L. W. Spatially Resolved Thermodynamics of the Partially Ionized Exciton Gas in GaAs. *Phys. Rev. Lett.* **2015**, *114*, 227402.

(15) Arkhipov, V. I.; Emelianova, E. V.; Bässler, H. Hot Exciton Dissociation in a Conjugated Polymer. *Phys. Rev. Lett.* **1999**, *82*, 1321-1324.

(16) Lee, J.; Vandewal, K.; Yost, S. R.; Bahlke, M. E.; Goris, L.; Baldo, M. A.; Manca, J. V.; Van Voorhis, T. Charge Transfer State Versus Hot Exciton Dissociation in Polymer−Fullerene Blended Solar Cells. *J. Am. Chem. Soc.* **2010**, *132*, 11878-11880.

(17) Grancini, G.; Maiuri, M.; Fazzi, D.; Petrozza, A.; Egelhaaf, H. J.; Brida, D.; Cerullo, G.; Lanzani, G. Hot exciton dissociation in polymer solar cells. *Nat. Mater.* **2013**, *12*, 29-33.

(18) Guo, J.; Ohkita, H.; Benten, H.; Ito, S. Near-IR Femtosecond Transient Absorption Spectroscopy of Ultrafast Polaron and Triplet Exciton Formation in Polythiophene Films with Different Regioregularities. *J. Am. Chem. Soc.* **2009**, *131*, 16869-16880.
